# Association of priori dietary patterns with metabolic syndrome and its components: a systematic review and meta-analysis

**DOI:** 10.3389/fnut.2026.1882985

**Published:** 2026-09-04

**Authors:** Mengzhen Fan, Linglun Hao, Qingjie Wu, Xianjing Wang, Guimei Lin, Zhe Zhang, Bizhen Gao

**Affiliations:** 1 College of Traditional Chinese Medicine, Fujian University of Traditional Chinese Medicine, Fuzhou, Fujian, China; 2 College of Integrative Medicine, Fujian University of Traditional Chinese Medicine, Fuzhou, Fujian, China

**Keywords:** dietary inflammatory index, Mediterranean diet, meta-analysis, metabolic syndrome, oxidative balance score, priori dietary patterns

## Abstract

**Background:**

Metabolic syndrome (MetS) poses an increasing threat to global health. Dietary patterns are recognized as modifiable factors related to MetS, but evidence on the associations of *a priori* dietary patterns with MetS remains inconsistent across studies.

**Methods:**

Embase, Web of Science, and PubMed were systematically searched up to March 20, 2025. Observational studies (cross-sectional, cohort, case–control) exploring the associations of *a priori* dietary patterns with MetS were included. The Joanna Briggs Institute tool and the Newcastle-Ottawa Scale were applied to assess the study quality. The certainty of the pooled evidence was assessed via the Grading of Recommendations Assessment, Development and Evaluation approach. A random-effects model was adopted to combine effect sizes.

**Results:**

Thirty-nine studies were incorporated. Categorical analyses showed that higher dietary inflammatory index (DII) was associated with higher prevalence of MetS (odds ratio [OR] [95% confidence interval (CI)]: 1.33 [1.23–1.43], *p* < 0.001). Higher Oxidative Balance Score (OBS) was associated with lower prevalence of MetS (0.48 [0.41–0.58], *p* < 0.001). High adherence to the Mediterranean diet (MD), Dietary Approaches to Stop Hypertension (DASH), Dietary Diversity Score (DDS), or Healthy Eating Index (HEI) showed no significant associations with MetS. Given the limited data for continuous variables, continuous analyses were only conducted for OBS and MD. These continuous analyses indicated that higher OBS and MD scores were associated with lower prevalence of MetS (for OBS: 0.94 [0.92–0.96], *p* < 0.001; for MD: 0.89 [0.83–0.96], *p* = 0.001).

**Conclusion:**

*A priori* dietary patterns are closely associated with MetS. Promoting anti-inflammatory, antioxidant-rich, and Mediterranean-style eating patterns may be associated with lower prevalence of MetS, which could provide potential implications for the prevention and management of MetS. However, causal inference remains limited, and further prospective studies and trials are needed.

**Systematic review registration:**

Unique identifier: CRD420251043513, https://www.crd.york.ac.uk/PROSPERO/view/CRD420251043513

## 1 Introduction

Metabolic syndrome (MetS) represents a disorder marked by a cluster of metabolic abnormalities. It is diagnosed when an individual presents with three or more of the following five core components: abnormal glucose homeostasis, low high-density lipoprotein (HDL) cholesterol, elevated blood pressure, abdominal adiposity, and high serum triglycerides (1). As a principal global health concern, the prevalence of MetS has risen rapidly over the last 20 years, exceeding 25% in the adult population worldwide. The rise is especially evident in low- and middle-income countries (2). MetS is a pivotal risk factor that directly contributes to chronic kidney disease, type 2 diabetes mellitus (T2DM), cardiovascular diseases, and other comorbidities, with high mortality and disability rates (3, 4). Furthermore, healthcare expenditures attributable to MetS and its complications are more than twice as high (5, 6), imposing a substantial economic burden and far-reaching social influences on global health systems. To mitigate this growing public health crisis, detecting modifiable determinants and formulating effective preventive strategies for MetS are imperative.

Lifestyle interventions are crucial for preventing and managing MetS (7), among which dietary intake is recognized as a pivotal modifiable factor influencing metabolic health (8). Early diet-related research on MetS has mainly centered on the influences of individual food components or nutrients. Nonetheless, the core of dietary intake resides not in single bioactive ingredients but in the combination and balance of various dietary components (9, 10). These investigations have failed to capture the synergistic and antagonistic effects of multiple dietary components in real-world dietary patterns, yielding inconsistent findings that are difficult to extrapolate to clinical and public health practice (11). To address this limitation, the focus of research has shifted toward the exploration of overall dietary patterns (12). Such a dietary approach reflects the cumulative effects of dietary intake, thereby furnishing a more realistic and comprehensive understanding of the relationship between metabolic health and diet (13). This has greatly advanced our knowledge of how dietary habits influence the occurrence of MetS and its constituent components.

Dietary patterns are generally classified as a posteriori and *a priori* patterns, among which *a priori* dietary patterns have emerged as an invaluable instrument in research on clinical and public health (14). Unlike a posteriori patterns, which are derived from statistical clustering of dietary data in specific study populations, *a priori* patterns are pre-defined based on established nutritional guidelines, theoretical dietary frameworks, and evidence-based dietary recommendations (15). A posteriori dietary patterns primarily answer the question of what people eat, whereas *a priori* dietary patterns address the extent to which people’s diets follow evidence-based dietary recommendations. The core strengths of *a priori* dietary patterns lie in their ease of assessment, clinical applicability, and direct translatability into population-based dietary recommendations (16). Nevertheless, such patterns may not fully capture population-specific dietary behaviors or culturally distinctive dietary characteristics. Although a posteriori dietary patterns may better reflect actual dietary behaviors within specific populations, their data-driven and population-specific nature may limit comparability across studies (17). Therefore, the present review focused on *a priori* dietary patterns to synthesize evidence on predefined and interpretable dietary indices related to MetS.

Although many studies have explored the link between *a priori* dietary patterns and MetS, the existing evidence remains inconsistent (18–20). For instance, some investigations have reported that high compliance with the Mediterranean diet (MD) exerts a protective effect against MetS (19, 21, 22), whereas others have failed to find a notable link (20, 23, 24). Furthermore, previous systematic reviews and meta-analyses merely focused on single dietary patterns (25) or individual dietary components (26), or broadly defined healthy diets (27), without explicitly distinguishing *a priori* and a posteriori dietary classification frameworks. Accordingly, a comprehensive systematic review and meta-analysis is needed to quantify the association of *a priori* dietary patterns with MetS and its individual components.

To address this gap, this meta-analysis aims to explore whether greater adherence to *a priori* dietary patterns in adults is associated with the prevalence of MetS and its components. The findings may offer references for developing evidence-informed dietary approaches relevant to the prevention and management of MetS.

## 2 Methods

The current analysis was implemented per the Preferred Reporting Items in Systematic Reviews and Meta-Analyses (PRISMA) guidelines (28). Its protocol was registered with the International Prospective Register of Systematic Reviews (CRD420251043513).

### 2.1 Search strategy

PubMed, Web of Science, and Embase were systematically searched up to March 20, 2025. The search combined keywords pertaining to dietary patterns and MetS, with the search strategy tailored to each database (Supplementary Table 1). In addition, the reference lists of all included studies and relevant reviews were manually screened to identify potentially eligible studies.

### 2.2 Eligibility criteria

The research question was formulated according to the PECOS framework to investigate whether greater adherence to *a priori* dietary patterns was associated with MetS and its components in adults. Specific items were listed as follows: (i) Population: patients (≥ 18 years old) diagnosed with MetS per the National Cholesterol Education Program Adult Treatment Panel III (NCEP-ATP III) criteria (29), without sex, race, nationality restriction; (ii) Exposure: high compliance with *a priori* dietary patterns (e.g., healthy eating index [HEI], dietary approaches to stop hypertension [DASH] diet, MD, and so on); (iii) Control: low compliance with *a priori* dietary patterns; (iv) Outcome: MetS and its components; (v) Study: observational studies (e.g., cross-sectional, cohort, and case–control).

Exclusion criteria comprised: (i) studies not reporting complete data for re-analysis, including effect estimates (odds ratio [OR] or relative risk [RR]), overall population, the number of cases, and 95% confidence intervals (CIs); (ii) studies that used a posteriori approach (e.g., cluster, factor, and principal component analyses) to derive dietary patterns; (iii) non-English investigations; (iv) animal studies, letters to the editor, reviews, case reports, and conference abstracts; (v) investigations that encompassed subjects with systematic diseases (tumor, lupus erythematosus, etc.) or pregnancy; (vi) studies with unavailable full texts.

### 2.3 Study selection and data extraction

All retrieved records were imported into NoteExpress (v4.1.0.10121, Aixiqin Lezhi Technology Co., Ltd) for literature management. The built-in deduplication function was used to remove duplicate records according to titles, authors, and DOIs. Subsequently, two independent researchers reviewed the remaining articles by title and abstract. Then, they utilized a customized Microsoft Excel form to extract data from all eligible studies independently. The extracted information comprised: author’s surname, study type, publication year, number of participants and cases, country, dietary patterns, assessment methods of dietary patterns, outcomes, confounding factors, and effect sizes of MetS and its components. Disagreements were adjudicated by a third researcher. No automated tools were applied during study screening or data extraction.

### 2.4 Methodological quality assessment

Two independent researchers assessed the quality of eligible studies. Specifically, a Joanna Briggs Institute (JBI) Critical Appraisal Tool was applied for cross-sectional studies (30), while the Newcastle-Ottawa Scale (NOS) was utilized for cohort and case–control studies (31). The JBI tool contains 8 items. Based on the information reported in each original study, each item was rated as ‘yes’, ‘no’, ‘unclear’, or ‘not applicable’. The NOS consists of 8 items, with a total score of 9. Each item was scored 1 if it met the criteria and 0 if it did not, except for one item which had an overall score of 2. The study quality was rated as low (0–3), moderate (4–6), and high (7–9), based on the total scores (32). Disagreements were adjudicated through a third researcher.

### 2.5 Certainty of evidence

The certainty of evidence for each pooled association of *a priori* dietary patterns with MetS and its components was assessed using the Grading of Recommendations Assessment, Development and Evaluation (GRADE) approach by two researchers independently (33). Disagreements were adjudicated by a third researcher. As all included studies were observational, the certainty of evidence initially started at low. The evidence was downgraded for risk of bias, inconsistency, indirectness, imprecision, or publication bias. It was upgraded when there was evidence of a large effect size, a dose–response gradient, or plausible residual confounding that would reduce the observed association. The final certainty of evidence was classified as high, moderate, low, or very low.

### 2.6 Statistical analysis

We could not convert effect estimates from various study designs into consistent RRs due to insufficient reported data. Therefore, the effect sizes of MetS associated with *a priori* dietary patterns were synthesized using OR with their corresponding 95% CIs, depending on the effect indicators reported in the eligible studies. Heterogeneity was examined via the I^2^ statistic. To ensure consistency of the analytical approach, a random-effects model was adopted for meta-analysis (34). Subgroup analysis was implemented to examine the possible influence of sex on the association of MetS with *a priori* dietary patterns. The same statistical models were applied in each subgroup to maintain comparability with the main analysis. The leave-one-out sensitivity analysis was implemented to appraise the stability of the pooled estimates (35). Publication bias was planned to be assessed using funnel plots and Egger’s test. Considering that each quantitative meta-analysis included fewer than 10 studies, both visual inspection of funnel plots and Egger’s test were limited by low statistical power and insufficient reliability. Therefore, funnel plots and Egger’s test were not performed (36). Statistical analyses were completed in Stata (v17.0, Stata Corporation).

## 3 Results

### 3.1 Study selection

**Figure 1:**
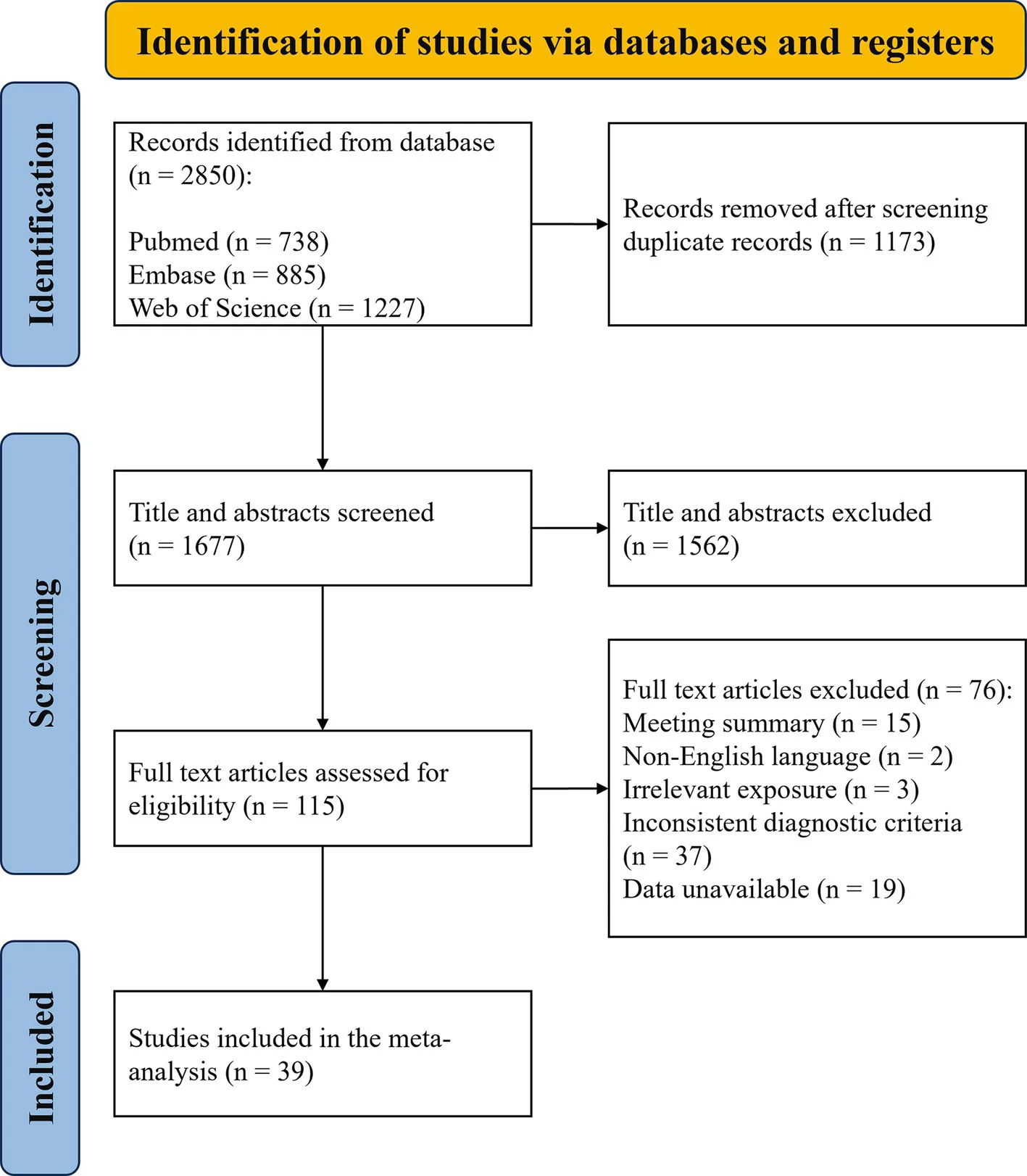
PRISMA flowchart for study selection: *a priori* dietary patterns and MetS.

The initial retrieval produced 2,850 records: Embase (*n* = 885), Web of Science (*n* = 1,227), PubMed (*n* = 738). After deduplication (*n* = 1,173 removed), 1,677 records were reviewed by title and abstract. Among these, 1,562 were excluded as irrelevant; the remaining 115 studies were read in full. A further 76 studies were subsequently eliminated because they were: non-English language (*n* = 2), conference abstracts (*n* = 15), irrelevant exposures (*n* = 3), failure to meet NCEP-ATP III diagnostic criteria (*n* = 37), and absence of analyzable data (*n* = 19). Finally, 39 studies (18, 19, 21, 23, 37–71) were incorporated into the meta-analysis. Figure 1 and Supplementary Table 2 illustrate the details.

### 3.2 Study characteristics

The 39 eligible studies encompassed cohort (*n* = 5), case–control (*n* = 1), and cross-sectional (*n* = 34) designs, including 1 with a mixed cohort and cross-sectional design. These studies were published between 2004 and 2025 across 11 countries, including Iran, Korea, the United States, China, and Spain. Individual sample sizes ranged from 97 to 157,812 participants. Sixteen studies focused only on MetS, while 23 investigated MetS and its components. Seventeen studies provided sex-stratified data for subgroup analysis. Only dietary patterns examined in ≥ 3 studies were eligible for quantitative synthesis. Six *a priori* dietary patterns met this criterion: DASH (6 studies), dietary diversity score (DDS, 4 studies), dietary inflammatory index (DII, 9 studies), HEI (6 studies), MD (9 studies), and oxidative balance score (OBS, 6 studies). Table 1 and Supplementary Table 3 present the details.

**Table 1:** General characteristics of the studies included in the meta-analysis on prior dietary patterns and metabolic syndrome and its components.

Author	Country	Study type	Sample size (Case)	Exposure	Diet assessment method	Control	Outcome	Effect value (95% CI)	Confounding factors	Study quality
Panagiotakos et al. (45)	Greece	Cross sectional	2,282 (453)	MD	Validated nutrient questionnaire	MD VS. non-MD	MetS	0.81 (0.68, 0.98)	Age, sex, smoking habits, educational status, several biochemical measurements, and their interactions	15
Azadbakht et al. (65)	Iran	Cross sectional	581 (91)	DDS	A 168-item semi-quantitative ffq	Q4 VS. Q1	MetS	0.77 (0.59, 0.93)	Age, total energy intake, energy from fat, use of blood pressure of estrogen medication, smoking, physical activity, and bmi	19
			581 (96)				Abdominal adiposity	0.96 (0.79, 1.41)		
			581 (248)				Low HDL-cholesterol	0.89 (0.59, 1.39)		
			581 (120)				High serum triglyceride	0.84 (0.69, 0.99)		
			581 (33)				Abnormal glucose homeostasis	0.78 (0.59, 0.98)		
			581 (56)				Elevated blood pressure	0.85 (0.58, 1.13)		
A’lvarez Leo’n et al. (69)	Spain	Cross sectional	578 (141)	MD	A semi-quantitative ffq	T3 VS. T1	MetS	1.37 (0.76, 2.46)	Sex, age, educational level, physical activity level, bmi, tobacco consumption, diet in the past 12 months, and energy intake	15
Tzima et al. (39)	Greece	Cross sectional	3,042 (607)	MD	A validated ffq (the epic-greek)	per 1 unit increase in diet score	MetS	0.93 (0.87, 1.01)	Age, sex, bmi, smoking habits, physical activity status	19
			1,528 (228)					Female 0.90 (0.88, 0.92)		
			1,514 (379)					Male 0.97 (0.95, 0.99)		
Gouveri et al. (21)	Greece	Cross sectional	2074 (538)	MD	A validated, extensive, semi-quantitative ffq	MD VS. non-MD	MetS	0.80 (0.65, 0.99)	Age, sex, smoking, light physical activity, serum levels of low-density lipoprotein cholesterol and γ-glutamyl transferase, diabetes mellitus, cvd, family history of hypertension, and/or hyperlipidemia	14
Huang et al. (58)	China	Cross sectional	1,071 (454)	VD	Household interview questions	VD VS. non VD	MetS	1.11 (0.82, 1.51)	Sex, smoking group, and exercise group	17
			1,071 (592)				Elevated blood pressure	1.02 (0.76, 1.39)		
Rizzo et al. (41)	US and Canada	Cross sectional	773 (265)	VD	A quantitative, self-administered ffq	VD VS. non VD	MetS	0.44 (0.30, 0.64)	Sex, ethnicity, smoking, alcohol intake, physical activity, and dietary energy intake	12
Saraf-Bank et al. (40)	Iran	Cross sectional	1,015 (167)	HEI-2010	A validated, self-administered, dish-based, semi-quantitative FFQ	Q4 VS. Q1	MetS	0.72 (0.50, 0.96)	Age, cigarette smoking, physical activity, socioeconomic status, current estrogen use, menopausal status, family history of diabetes, stroke, bmi	18
			1,015 (341)				Abdominal adiposity	0.79 (0.59, 0.99)		
			1,015 (416)				Low HDL-cholesterol	0.75 (0.68, 0.89)		
			1,015 (145)				High serum triglyceride	0.65 (0.38, 0.92)		
			1,015 (61)				Abnormal glucose homeostasis	0.80 (0.51, 1.11)		
			1,015 (228)				Elevated blood pressure	0.69 (0.51, 0.80)		
Gholizadeh et al. (63)	Iran	Cross sectional	300 (113)	DDS	A valid semi quantitative ffq with 168 items	T4 VS. T1	MetS	0.21 (0.08, 0.57)	Total energy, sex, age, wc, bmi	19
Kang et al. (55)	Korea	Cross sectional	6,826 (2204)	DASH	A qualitative ffq and a 24-h recall	per 1 unit increase in diet score	MetS	0.98 (0.96, 0.99)	Age, body mass index, income, smoking habits, alcohol intake, physical activity, education level, and total calorie intake	18
Kim et al. (52)	Korea	Cross sectional	9,291 (2009)	DII	A 24-h dietary recall	Q4 VS. Q1	MetS	1.31 (1.07, 1.61)	Age, bmi, education, alcohol consumption, smoking, physical activity, and total calorie intake	19
			5,609 (966)					Female 1.22 (0.91, 1.64)		
			3,682 (1043)					Male 1.40 (1.06, 1.85)		
			9,291 (2054)				Abdominal adiposity	1.22 (0.93, 1.59)		
			5,609 (1044)					Female 1.35 (0.94, 1.94)		
			3,682 (1010)					Male 1.07 (0.72, 1.61)		
			9,291 (2994)				Low HDL-cholesterol	0.88 (0.75, 1.02)		
			5,609 (2092)					Female 0.85 (0.71, 1.04)		
			3,682 (902)					Male 0.93 (0.72, 1.21)		
			9,291 (2549)				High serum triglyceride	1.15 (0.97, 1.36)		
			5,609 (1060)					Female 1.07 (0.84, 1.38)		
			3,682 (1489)					Male 1.22 (0.97, 1.53)		
			9,291 (2676)				Abnormal glucose homeostasis	1.11 (0.81, 1.50)		
			5,609 (1292)					Female 0.95 (0.77, 1.18)		
			3,682 (1384)					Male 1.30 (1.02, 1.65)		
			9,291 (2764)				Elevated blood pressure	1.12 (0.94, 1.33)		
			5,609 (1335)					Female 1.10 (0.87, 1.38)		
			3,682 (1429)					Male 1.14 (0.88, 1.46)		
Nikniaz et al. (47)	Iran	Cross sectional	606 (208)	DII	A validated quantitative FFQ	Q4 VS. Q1	MetS	2.26 (1.03, 4.92)	Smoking status (smoker and non-smoker), physical activity (low, moderate, high), sex, age, bmi	18
			606 (292)				Abdominal adiposity	0.86 (0.39, 1.91)		
			606 (NA)				Low HDL-cholesterol	0.83 (0.44, 1.55)		
			606 (267)				High serum triglyceride	1.31 (0.66, 2.58)		
			606 (101)				Abnormal glucose homeostasis	2.56 (1.01, 7.05)		
			606 (NA)				Elevated blood pressure	1.18 (0.47, 2.96)		
Ghorabi et al. (61)	Iran	Cross sectional	396 (192)	DASH	A validated 147-item semi-quantitative FFQ	T3 VS. T1	MetS	0.28 (0.14, 0.54)	Age, sex, energy intake, marital status, physical activity, educational status, smoking, economic status, and supplementation, bmi	16
			396 (133)				Abdominal adiposity	1.16 (0.48, 2.82)		
			396 (132)				Low HDL-cholesterol	0.51 (0.25, 1.01)		
			396 (133)				High serum triglyceride	0.53 (0.28, 1.00)		
			396 (141)				Abnormal glucose homeostasis	0.65 (0.36, 1.17)		
			396 (133)				Elevated blood pressure	0.12 (0.05, 0.29)		
Phillips et al. (42)	Ireland	Cross sectional	1,493 (317)	DASH	A modified version of the self-completed EPIC FFQ	Q4 VS. Q1	MetS	0.52 (0.28, 0.94)	Age, sex, bmi, physical activity, smoking status, alcohol consumption, dietary energy intake, anti-inflammatory and lipid-lowering medication use	17
Shin et al. (23)	Korea	Cohort	5,549 (1891)	DASH	A semi-quantitative FFQ with 103 items which was developed and validated among Korean adults	Q5 VS. Q1	MetS	0.97 (0.83, 1.13)	Age, total energy intake, income level, education level, physical activity, smoking, drinking	8
			2,744 (943)					Female 0.90 (0.72, 1.13)		
			2,805 (948)					Male 1.04 (0.84, 1.29)		
			5,549 (1891)	MD				0.94 (0.79, 1.11)		
			2,744 (943)					Female 1.02 (0.80, 1.29)		
			2,805 (948)					Male 0.86 (0.68, 1.09)		
Abdollahzad et al. (71)	Iran	Cross sectional	6,538 (1901)	DII	The validated national Iranian FFQ	Q4 VS. Q1	MetS	1.34 (1.01, 1.77)	Age, sex, education, marital status, economic status, smoking, physical activity level, total energy intake, bmi	17
			6,538 (2572)				Abdominal adiposity	1.17 (0.91, 1.52)		
			6,538 (1622)				Low HDL-cholesterol	1.24 (1.01, 1.53)		
			6,538 (1857)				High serum triglyceride	1.29 (1.02, 1.62)		
			6,538 (582)				Abnormal glucose homeostasis	1.57 (1.10, 2.24)		
			6,538 (NA)				Elevated blood pressure	1.56 (0.97, 2.50)		
Ariya et al. (66)	Iran	Cross sectional	10,017 (2219)	DII	A 125-item FFQ	Q4 VS. Q1	MetS	1.37 (1.18, 1.59)	Bmi and age	17
			10,017 (4465)				Abdominal adiposity	2.70 (2.31, 3.16)		
			10,017 (4216)				Low HDL-cholesterol	1.29 (1.15, 1.44)		
			10,017 (2822)				High serum triglyceride	0.77 (0.68, 0.88)		
			10,017 (1919)				Abnormal glucose homeostasis	1.16 (1.00, 1.34)		
			10,017 (2323)				Elevated blood pressure	1.28 (1.11, 1.47)		
Canto-Osorio et al. (64)	Mexico	Cohort	399 (165)	DII	Using a previously validated 116-item semi quantitative FFQ	Q4 VS. Q1	MetS	1.99 (1.03, 3.85)	Age, sex, educational level, smoking status, physical activity, sleep time, and energy intake	6
			275 (NA)				Abdominal adiposity	2.68 (1.06, 6.79)		
			90 (NA)				Low HDL-cholesterol	1.27 (0.31, 5.19)		
			311 (NA)				High serum triglyceride	2.28 (1.13, 4.57)		
			374 (NA)				Abnormal glucose homeostasis	1.33 (0.68, 2.58)		
			382 (NA)				Elevated blood pressure	2.22 (1.03, 4.77)		
Ghorabi et al. (62)	Iran	Cross sectional	404 (148)	DII	A validated 147-item food FFQ	T3 VS. T1	MetS	0.92 (0.48, 1.76)	Age, sex, energy intake, marital status, physical activity, education status, smoking, economic status, supplementation, bmi	17
			404 (NA)				Abdominal adiposity	1.11 (0.45, 2.71)		
			404 (NA)				Low HDL-cholesterol	2.71 (1.34, 5.47)		
			404 (NA)				High serum triglyceride	0.71 (0.38, 1.32)		
			404 (NA)				Abnormal glucose homeostasis	0.87 (0.48, 1.57)		
			404 (NA)				Elevated systolic blood pressure	0.47 (0.22, 1.93)		
			404 (NA)				Elevated diastolic blood pressure	0.45 (0.21, 1.10)		
Khan et al. (54)	Korea	Cohort	157,812 (3507)	DII	A semi-quantitative FFQ	Q5 VS. Q1	MetS	1.31 (1.15, 1.49)	Sex, age, smoking, alcohol drinking, physical activity, bmi, family history of diabetes mellitus, family history of hypertension, and energy intake	8
			104,508 (2112)					Female 1.43 (1.21, 1.69)		
			53,304 (1395)					Male 1.14 (0.93, 1.39)		
			157,812 (5885)				Abdominal adiposity	1.37 (1.24, 1.51)		
			104,508 (NA)					Female 1.42 (1.26, 1.61)		
			53,304 (NA)					Male 1.28 (1.08, 1.52)		
			157,812 (3937)				Low HDL-cholesterol	1.63 (1.44, 1.84)		
			104,508 (NA)					Female 1.64 (1.41, 1.90)		
			53,304 (NA)					Male 1.59 (1.27, 1.99)		
			157,812 (8964)				High serum triglyceride	1.24 (1.14, 1.35)		
			104,508 (NA)					Female 1.30 (1.18, 1.44)		
			53,304 (NA)					Male 1.11 (0.96, 1.28)		
			157,812 (11337)				Abnormal glucose homeostasis	1.18 (1.09, 1.26)		
			104,508 (NA)					Female 1.30 (1.18, 1.43)		
			53,304 (NA)					Male 1.01 (0.90, 1.13)		
			157,812 (10148)				Elevated blood pressure	1.24 (1.15, 1.34)		
			104,508 (NA)					Female 1.29 (1.17, 1.41)		
			53,304 (NA)					Male 1.17 (1.03, 1.32)		
			157,812 (3507)			per 1 unit increase	MetS	1.02 (1.01, 1.04)		
			104,508 (2112)					Female 1.04 (1.02, 1.07)		
			53,304 (1395)					Male 0.98 (0.96, 1.01)		
			157,812 (5885)				Abdominal adiposity	1.06 (1.04, 1.07)		
			104,508 (NA)					Female 1.06 (1.03, 1.08)		
			53,304 (NA)					Male 1.06 (1.03, 1.09)		
			157,812 (3937)				Low HDL-cholesterol	1.08 (1.06, 1.11)		
			104,508 (NA)					Female 1.09 (1.06, 1.12)		
			53,304 (NA)					Male 1.06 (1.02, 1.11)		
			157,812 (8964)				High serum triglyceride	1.03 (1.01, 1.04)		
			104,508 (NA)					Female 1.03 (1.02, 1.05)		
			53,304 (NA)					Male 1.01 (0.98, 1.03)		
			157,812 (11337)				Abnormal glucose homeostasis	1.02 (1.01, 1.03)		
			104,508 (NA)					Female 1.04 (1.02, 1.05)		
			53,304 (NA)					Male 0.99 (0.97, 1.01)		
			157,812 (10148)				Elevated blood pressure	1.03 (1.02, 1.04)		
			104,508 (NA)					Female 1.04 (1.02, 1.05)		
			53,304 (NA)					Male 1.02 (1.00, 1.04)		
Kim et al. (53)	Korea	Cohort	5,646 (2583)	PDI	A validated 106-item semi-quantitative FFQ	Q5 VS. Q1	MetS	0.96 (0.82, 1.04)	Age, sex, total energy intake, education, physical activity, smoking status, alcohol intake, and bmi	9
						Per SD		0.96 (0.92, 1.00)		
			5,646 (NA)			Q5 VS. Q1	Abdominal adiposity	0.95 (0.82, 1.09)		
			5,646 (NA)				Low HDL-cholesterol	1.13 (0.99, 1.27)		
			5,646 (NA)				High serum triglyceride	0.98 (0.85, 1.13)		
			5,646 (NA)				Abnormal glucose homeostasis	0.80 (0.70, 0.92)		
			5,646 (NA)				Elevated blood pressure	1.01 (0.87, 1.16)		
			5,646 (2583)	hPDI		Q5 VS. Q1	MetS	0.97 (0.86, 1.10)		
						Per SD		0.96 (0.93, 1.00)		
			5,646 (NA)			Q5 VS. Q1	Abdominal adiposity	1.04 (0.89, 1.20)		
			5,646 (NA)				Low HDL-cholesterol	1.08 (0.95, 1.22)		
			5,646 (NA)				High serum triglyceride	1.00 (0.87, 1.15)		
			5,646 (NA)				Abnormal glucose homeostasis	1.01 (0.88, 1.16)		
			5,646 (NA)				Elevated blood pressure	0.93 (0.80, 1.07)		
			5,646 (2583)	uPDI		Q5 VS. Q1	MetS	1.44 (1.26, 1.64)		
						Per SD		1.15 (1.11, 1.21)		
			5,646 (NA)			Q5 VS. Q1	Abdominal adiposity	1.46 (1.25, 1.71)		
			5,646 (NA)				Low HDL-cholesterol	1.25 (1.09, 1.43)		
			5,646 (NA)				High serum triglyceride	1.26 (1.08, 1.46)		
			5,646 (NA)				Abnormal glucose homeostasis	1.06 (0.91, 1.24)		
			5,646 (NA)				Elevated blood pressure	1.24 (1.06, 1.45)		
			5,646 (2583)	Pro-vegetarian Diet Index		Q5 VS. Q1	MetS	1.10 (0.97, 1.25)		
						Per SD		1.02 (0.98, 1.06)		
			5,646 (NA)			Q5 VS. Q1	Abdominal adiposity	0.99 (0.84, 1.15)		
			5,646 (NA)				Low HDL-cholesterol	1.12 (0.98, 1.28)		
			5,646 (NA)				High serum triglyceride	1.02 (0.88, 1.18)		
			5,646 (NA)				Abnormal glucose homeostasis	0.93 (0.80, 1.08)		
			5,646 (NA)				Elevated blood pressure	0.99 (0.85, 1.16)		
Amini et al. (67)	Iran	Cross sectional	178 (95)	PDI	A valid and reliable semi-quantitative FFQ	T3 VS. T1	MetS	1.05 (0.49, 2.27)	Physical activity, smoking, income, age, sex, marital status, diabetes, hypertension, dyslipidemia, bmi, energy	18
				hPDI				0.79 (0.37, 1.68)		
				uPDI				0.77 (0.35, 1.69)		
Noruzi et al. (46)	Iran	Cross sectional	847 (258)	OBS	A semi-quantitative FFQ with 168 items	T3 VS. T1	MetS	0.71 (0.48, 1.03)	Age, sex, energy intake, occupation, and educational level	18
			847 (NA)				Abdominal adiposity	0.55 (0.38, 0.81)		
			847 (NA)				Low HDL-cholesterol	1.07 (0.70, 1.62)		
			847 (NA)				High serum triglyceride	0.78 (0.56, 1.14)		
			847 (NA)				Abnormal glucose homeostasis	0.72 (0.48, 1.10)		
			847 (NA)				Elevated systolic blood pressure	0.91 (0.59, 1.40)		
			847 (NA)				Elevated diastolic blood pressure	0.64 (0.41, 0.99)		
Zadeh et al. (18)	Iran	Cross sectional	2,221 (640)	DASH	The validated FFQ containing 178 food items and 551 questions	Q5 VS. Q1	MetS	0.81 (0.50, 1.30)	Age, sex, energy intake, smoking status, marriage status, physical activity, education level, job status, house status, number of family members, house area, ethnicity, disease history, bmi	16
			NA (NA)					Female 0.50 (0.27, 0.95)		
			NA (NA)					Male 2.26 (0.87, 2.89)		
			2,221 (979)				Abdominal adiposity	0.71 (0.39, 1.28)		
			NA (NA)					Female 0.34 (0.15, 0.77)		
			NA (NA)					Male 1.45 (0.59, 3.58)		
			2,221 (777)				Low HDL-cholesterol	0.80 (0.52, 1.24)		
			NA (NA)					Female 0.68 (0.38, 1.21)		
			NA (NA)					Male 0.97 (0.48, 1.96)		
			2,221 (1359)				High serum triglyceride	1.21 (0.78, 1.88)		
			NA (NA)					Female 1.31 (0.67, 2.54)		
			NA (NA)					Male 1.12 (0.61, 2.06)		
			2,221 (686)				Abnormal glucose homeostasis	0.76 (0.42, 1.36)		
			NA (NA)					Female 0.74 (0.33, 1.64)		
			NA (NA)					Male 1.06 (0.99, 1.13)		
			2,221 (1544)				Elevated blood pressure	1.41 (0.88, 2.26)		
			NA (NA)					Female 1.08 (0.54, 2.18)		
			NA (NA)					Male 1.70 (0.86, 3.38)		
			2,221 (640)	MD			MetS	1.09 (0.60, 1.98)		
			NA (NA)					Female 0.93 (0.43, 1.99)		
			NA (NA)					Male 1.57 (0.55, 4.47)		
			2,221 (979)				Abdominal adiposity	0.64 (0.33, 1.26)		
			NA (NA)					Female 0.50 (0.19, 1.32)		
			NA (NA)					Male 0.89 (0.31, 2.54)		
			2,221 (777)				Low HDL-cholesterol	1.05 (0.62, 1.79)		
			NA (NA)					Female 0.94 (0.46, 1.93)		
			NA (NA)					Male 1.16 (0.51, 2.62)		
			2,221 (1359)				High serum triglyceride	1.09 (0.66, 1.80)		
			NA (NA)					Female 1.51 (0.70, 3.26)		
			NA (NA)					Male 0.86 (0.43, 1.72)		
			2,221 (686)				Abnormal glucose homeostasis	0.88 (0.44, 1.77)		
			NA (NA)					Female 0.70 (0.27, 1.77)		
			NA (NA)					Male 1.40 (0.43, 4.56)		
			2,221 (1544)				Elevated blood pressure	1.25 (0.71, 2.19)		
			NA (NA)					Female 1.01 (0.42, 2.42)		
			NA (NA)					Male 1.53 (0.70, 3.32)		
Zadeh et al. (18)	Iran	Cross sectional	2,326 (526)	HEI-2015	The validated FFQ	Q5 VS. Q1	MetS	1.08 (0.66, 1.78)	Age, energy intake, marriage, physical activity, education level, smoking, job status, house status, number of family members, house area, ethnicity, disease history, bmi	16
			NA (NA)					Female 0.93 (0.49, 1.76)		
			NA (NA)					Male 1.82 (0.74, 4.45)		
			2,326 (NA)				Abdominal adiposity	1.08 (0.60, 1.92)		
			2,326 (NA)				Low HDL-cholesterol	1.11 (0.72, 1.72)		
			2,326 (NA)				High serum triglyceride	0.89 (0.58, 1.37)		
			2,326 (NA)				Abnormal glucose homeostasis	0.89 (0.56, 1.40)		
			NA (NA)					Female 0.67 (0.31, 1.48)		
			NA (NA)					Male 0.64 (0.27, 1.53)		
			2,326 (NA)				Elevated blood pressure	1.37 (0.82, 2.30)		
Kudsee et al. (70)	Luxembourg	Cross sectional	1,344 (367)	MD	A validated ffq with 174 items	Q4 VS. Q1	MetS	0.92 (0.77, 1.08)	Age group, sex, and all sociodemographic variables, and selected anthropometric variables such as (education, job, income, marital status, physical activity, smoking, total energy intake)	18
						per 1 unit increase in diet score		0.93 (0.81, 1.05)		
				AHEI		Q4 VS. Q1		0.99 (0.81, 1.22)		
						per 1 unit increase in diet score		1.00 (0.97, 1.02)		
Li et al. (50)	China	Cross sectional	1936 (828)	DII	A validated semi-quantitative ffq	Q4 VS. Q1	MetS	1.28 (1.08, 1.56)	Age, sex, educational level, residence, employment status, tobacco smoking, and physical activity	16
			1,200 (NA)					Female 1.36 (1.08, 1.57)		
			736 (NA)					Male 1.65 (1.08, 2.06)		
			1936 (786)				Abdominal adiposity	1.59 (1.18, 2.06)		
			1,200 (NA)					Female 1.63 (1.18, 2.02)		
			736 (NA)					Male 1.52 (1.18, 1.96)		
			1936 (773)				Low HDL-cholesterol	1.08 (0.73, 1.26)		
			1,200 (NA)					Female 1.19 (0.98, 1.75)		
			736 (NA)					Male 1.15 (0.92, 1.61)		
			1936 (671)				High serum triglyceride	1.39 (1.12, 1.65)		
			1,200 (NA)					Female 1.39 (1.05, 1.72)		
			736 (NA)					Male 1.37 (1.02, 1.68)		
			1936 (975)				Abnormal glucose homeostasis	1.23 (1.05, 1.49)		
			1,200 (NA)					Female 1.68 (1.09, 2.06)		
			736 (NA)					Male 1.76 (1.13, 2.15)		
			1936 (1270)				Elevated blood pressure	1.28 (1.09, 1.51)		
			1,200 (NA)					Female 1.35 (1.13, 1.62)		
			736 (NA)					Male 1.36 (1.08, 1.82)		
Jovanovic et al. (57)	US	Cross sectional	9,544 (4264)	Healthy PBF	Two 24-h dietary recalls	a 1-unit increase in daily servings	MetS	0.96 (0.93, 0.99)	Sex, age, income, and ethnicity	14
Kahrizsangi et al. (68)	Iran	Cross sectional	2,225 (607)	HEI-2015	The validated, self-administered, semi-quantitative FFQ containing 168 food items	T3 VS. T1	MetS	0.65 (0.52, 0.82)	Age, bmi, physical activity, smoking status, marital status, menopausal status, education status, and use of medications	16
			1,038 (363)					Female 0.88 (0.64, 1.21)		
			1,187 (244)					Male 0.48 (0.33, 0.68)		
			2,225 (350)				Abdominal adiposity	0.88 (0.63, 1.23)		
			1,038 (NA)					Female 0.60 (0.31, 1.15)		
			1,187 (NA)					Male 1.51 (0.76, 3.02)		
			2,225 (437)				Low HDL-cholesterol	1.01 (0.81, 1.25)		
			1,038 (NA)					Female 1.16 (0.84, 1.61)		
			1,187 (NA)					Male 0.89 (0.66, 1.20)		
			2,225 (243)				High serum triglyceride	0.72 (0.57, 0.90)		
			1,038 (NA)					Female 0.59 (0.42, 0.84)		
			1,187 (NA)					Male 0.83 (0.61, 1.13)		
			2,225 (170)				Abnormal glucose homeostasis	0.81 (0.63, 1.03)		
			1,038 (NA)					Female 0.82 (0.57, 1.18)		
			1,187 (NA)					Male 0.78 (0.55, 1.10)		
			2,225 (50)				Elevated blood pressure	0.58 (0.38, 0.90)		
			1,038 (NA)					Female 0.51 (0.23, 1.10)		
			1,187 (NA)					Male 0.46 (0.26, 0.82)		
Park et al. (44)	Korea	Cohort	5,807 (2176)	OBS	24-h food recalls	T3 VS. T1	MetS	0.60 (0.53, 0.67)	Age, education level, monthly household income, total energy intake, mean blood pressure, whole blood white blood cell count, fasting plasma glucose, and serum total cholesterol levels based on characteristics for each population	9
			2,886 (880)					Female 0.63 (0.55, 0.73)		
			2,921 (1296)					Male 0.56 (0.48, 0.65)		
			5,807 (2176)			per 1 unit increase in diet score		0.90 (0.88, 0.92)		
			2,886 (880)					Female 0.90 (0.88, 0.93)		
			2,921 (1296)					Male 0.90 (0.88, 0.92)		
		Cross sectional	2,735 (719)			T3 VS. T1	MetS	0.38 (0.29, 0.51)	Age, education level, monthly household income, total energy intake, mean blood pressure, whole blood white blood cell count, fasting plasma glucose, and serum total cholesterol levels based on characteristics for each population	17
			1,550 (358)					Female 0.34 (0.23, 0.50)		
			1,185 (361)					Male 0.44 (0.29, 0.65)		
			2,735 (719)			per 1 unit increase in diet score		0.84 (0.77, 0.91)		
			1,550 (358)					Female 0.80 (0.75, 0.86)		
			1,185 (361)					Male 0.87 (0.81, 0.92)		
Xu et al. (28)	US	Cross sectional	11,171 (3622)	OBS	At least one 24-h dietary recall	Q4 VS. Q1	MetS	0.42 (0.33, 0.53)	Age, sex, race/ethnicity, educational level, pir, dietary energy intake	15
						per 1 unit increase in diet score		0.95 (0.94, 0.96)		
			5,085 (NA)					Female 0.94 (0.93, 0.96)		
			6,086 (NA)					Male 0.95 (0.94, 0.96)		
			11,171 (3622)			per SD		0.68 (0.62, 0.74)		
			11,171 (5512)			Q4 VS. Q1	Abdominal adiposity	0.26 (0.22, 0.31)		
						per 1 unit increase in diet score		0.93 (0.92, 0.93)		
			11,171 (3941)			Q4 VS. Q1	Low HDL-cholesterol	0.54 (0.44, 0.67)		
						per 1 unit increase in diet score		0.96 (0.95, 0.97)		
			11,171 (4420)			Q4 VS. Q1	High serum triglyceride	0.54 (0.44, 0.67)		
						per 1 unit increase in diet score		0.96 (0.95, 0.97)		
			11,171 (2710)			Q4 VS. Q1	Abnormal glucose homeostasis	0.75 (0.59, 0.96)		
						per 1 unit increase in diet score		0.98 (0.97, 0.99)		
			11,171 (3861)			Q4 VS. Q1	Elevated blood pressure	0.55 (0.42, 0.71)		
						per 1 unit increase in diet score		0.96 (0.95, 0.97)		
Gómez-Sánchez et al. (19)	Spain	Cross sectional	3,417 (1423)	MD	A 14-item questionnaire, which was validated in Spain	MD VS. non-MD	MetS	0.56 (0.47, 0.65)	Age, sex, and consumption of antihypertensive drugs, hypoglycemic, and lipid-lowering agents	17
			1,468 (663)					Female 0.48 (0.38, 0.61)		
			1849 (760)					Male 0.64 (0.52, 0.79)		
			3,417 (1966)				Abdominal adiposity	0.74 (0.64, 0.86)		
			1,468 (1018)					Female 0.70 (0.55, 0.88)		
			1849 (948)					Male 0.78 (0.65, 0.95)		
			3,417 (973)				Low HDL-cholesterol	1.70 (1.44, 2.01)		
			1,468 (546)					Female 1.72 (1.37, 2.15)		
			1849 (427)					Male 1.66 (1.30, 2.12)		
			3,417 (989)				High serum triglyceride	0.64 (0.55, 0.75)		
			1,468 (354)					Female 0.77 (0.60, 0.99)		
			1849 (635)					Male 0.61 (0.49, 0.75)		
			3,417 (1294)				Abnormal glucose homeostasis	0.62 (0.51, 0.74)		
			1,468 (502)					Female 0.58 (0.44, 0.78)		
			1849 (793)					Male 0.64 (0.50, 0.82)		
			3,417 (2561)				Elevated blood pressure	0.63 (0.52, 0.77)		
			1,468 (998)					Female 0.66 (0.49, 0.88)		
			1849 (1563)					Male 0.62 (0.48, 0.81)		
Jowshan et al. (56)	Iran	Cross sectional	97 (58)	HEI-2015	FFQ	per 1 unit increase in diet score	MetS	0.93 (0.88, 0.99)	Age, bmi, daily energy intake, physical activity level	19
Li et al. (51)	US	Cross sectional	16,850 (4857)	OBS	24-h dietary recall interviews	Q4 VS. Q1	MetS	0.55 (0.47, 0.64)	Age, sex, race, marital status, education level, energy intake, and crp level	16
						per 1 unit increase in diet score		0.96 (0.95, 0.97)		
			7,862 (NA)			Q4 VS. Q1		Female 0.57 (0.46, 0.72)		
			8,988 (NA)					Male 0.50 (0.40, 0.63)		
			16,850 (NA)			Q4 VS. Q1	Abdominal adiposity	0.61 (0.54, 0.69)		
						per 1 unit increase in diet score		0.95 (0.94, 0.96)		
			7,862 (NA)			Q4 VS. Q1		Female 0.48 (0.39, 0.60)		
			8,988 (NA)					Male 0.40 (0.32, 0.51)		
			16,850 (NA)			Q4 VS. Q1	Low HDL-cholesterol	0.60 (0.50, 0.70)		
						per 1 unit increase in diet score		0.97 (0.96, 0.98)		
			7,862 (NA)			Q4 VS. Q1		Female 0.53 (0.42, 0.68)		
			8,988 (NA)					Male 0.67 (0.53, 0.84)		
			16,850 (NA)			Q4 VS. Q1	High serum triglyceride	0.68 (0.57, 0.82)		
						per 1 unit increase in diet score		0.98 (0.97, 0.99)		
			7,862 (NA)			Q4 VS. Q1		Female 0.68 (0.53, 0.88)		
			8,988 (NA)					Male 0.68 (0.53, 0.86)		
			16,850 (NA)			Q4 VS. Q1	Abnormal glucose homeostasis	0.74 (0.62, 0.88)		
						per 1 unit increase in diet score		0.97 (0.96, 0.98)		
			7,862 (NA)			Q4 VS. Q1		Female 0.66 (0.52, 0.84)		
			8,988 (NA)					Male 0.79 (0.63, 1.00)		
			16,850 (NA)			Q4 VS. Q1	Elevated blood pressure	0.69 (0.58, 0.83)		
						per 1 unit increase in diet score		0.98 (0.97, 0.99)		
			7,862 (NA)			Q4 VS. Q1		Female 0.69 (0.51, 0.93)		
			8,988 (NA)					Male 0.69 (0.55, 0.85)		
Lu et al. (49)	US	Cross sectional	10,025 (3005)	OBS	24-h food recalls	Q4 VS. Q1	MetS	0.42 (0.33, 0.53)	Age, sex, race, pir, marital status, education level, energy intake, caffeine intake, and sleep problems	16
						per 1 unit increase in diet score		0.95 (0.94, 0.96)		
			4,864 (1469)					Female 0.96 (0.95, 0.97)		
			5,161 (1536)					Male 0.97 (0.95, 0.98)		
			10,025 (5593)			Q4 VS. Q1	Abdominal adiposity	0.31 (0.25, 0.38)		
						per 1 unit increase in diet score		0.93 (0.92, 0.94)		
			10,025 (2657)			Q4 VS. Q1	Low HDL-cholesterol	0.52 (0.41, 0.67)		
						per 1 unit increase in diet score		0.96 (0.95, 0.97)		
			10,025 (3640)			Q4 VS. Q1	High serum triglyceride	0.54 (0.44, 0.67)		
						per 1 unit increase in diet score		0.96 (0.95, 0.97)		
			10,025 (2547)			Q4 VS. Q1	Abnormal glucose homeostasis	0.64 (0.51, 0.82)		
						per 1 unit increase in diet score		0.98 (0.96, 0.99)		
			10,025 (3203)			Q4 VS. Q1	Elevated blood pressure	0.62 (0.47, 0.83)		
						per 1 unit increase in diet score		0.97 (0.96, 0.99)		
Pashaei et al. (43)	Iran	Cross sectional	5,206 (895)	DASH	A validated 134-item semi-quantitative FFQ	per 1 unit increase in diet score	MetS	0.94 (0.93, 0.95)	Age, sex, physical activity, energy, wsi, and smoking cigarettes	17
			5,206 (NA)				Abdominal adiposity	1.00 (1.00, 1.01)		
			5,206 (NA)				Low HDL-cholesterol	0.98 (0.97, 0.98)		
			5,206 (NA)				High serum triglyceride	0.98 (0.97, 0.98)		
			5,206 (NA)				Abnormal glucose homeostasis	1.00 (0.99, 1.01)		
			5,206 (NA)				Elevated systolic blood pressure	1.01 (1.00, 1.02)		
			5,206 (NA)				Elevated diastolic blood pressure	0.98 (0.97, 1.00)		
			5,206 (895)	DII			MetS	1.22 (1.11, 1.34)		
			5,206 (NA)				Abdominal adiposity	0.94 (0.90, 0.99)		
			5,206 (NA)				Low HDL-cholesterol	1.01 (0.95, 1.07)		
			5,206 (NA)				High serum triglyceride	0.98 (0.93, 1.03)		
			5,206 (NA)				Abnormal glucose homeostasis	1.00 (0.95, 1.05)		
			5,206 (NA)				Elevated systolic blood pressure	1.01 (0.93, 1.10)		
			5,206 (NA)				Elevated diastolic blood pressure	1.05 (0.95, 1.16)		
			5,206 (895)	MD			MetS	0.85 (0.81, 0.89)		
			5,206 (NA)				Abdominal adiposity	1.00 (0.97, 1.03)		
			5,206 (NA)				Low HDL-cholesterol	0.95 (0.91, 0.99)		
			5,206 (NA)				High serum triglyceride	0.96 (0.93, 1.07)		
			5,206 (NA)				Abnormal glucose homeostasis	0.92 (0.88, 0.95)		
			5,206 (NA)				Elevated systolic blood pressure	1.00 (0.94, 1.06)		
			5,206 (NA)				Elevated diastolic blood pressure	0.98 (0.91, 1.05)		
			5,206 (895)	MIND			MetS	0.84 (0.80, 0.88)		
			5,206 (NA)				Abdominal adiposity	1.02 (0.99, 1.06)		
			5,206 (NA)				Low HDL-cholesterol	0.95 (0.91, 0.99)		
			5,206 (NA)				High serum triglyceride	0.99 (0.95, 1.03)		
			5,206 (NA)				Abnormal glucose homeostasis	1.01 (0.97, 1.04)		
			5,206 (NA)				Elevated systolic blood pressure	1.09 (1.03, 1.15)		
			5,206 (NA)				Elevated diastolic blood pressure	0.95 (0.88, 1.01)		
			5,206 (895)	DQI			MetS	0.95 (0.94, 0.96)		
			5,206 (NA)				Abdominal adiposity	0.99 (0.98, 0.99)		
			5,206 (NA)				Low HDL-cholesterol	0.98 (0.97, 0.99)		
			5,206 (NA)				High serum triglyceride	0.99 (0.98, 1.00)		
			5,206 (NA)				Abnormal glucose homeostasis	0.99 (0.98, 1.00)		
			5,206 (NA)				Elevated systolic blood pressure	1.00 (0.99, 1.02)		
			5,206 (NA)				Elevated diastolic blood pressure	0.99 (0.98, 1.01)		
Htay et al. (59)	Japan	Cross sectional	75,332 (11376)	DDS	A short FFQ	Q4 VS. Q1	MetS	0.91 (0.84, 0.98)	Age, sex, years of education, smoking status, drinking status, regular physical exercise, and site	15
Mohtashami et al. (48)	Iran	case control	350 (175)	DDS	A validated 168-item FFQ	Q4 VS. Q1	MetS	1.30 (0.64, 2.64)	Age, sex, energy intake, job, income, education, and marital status	6
				AHEI				0.85 (0.43, 1.70)		
Zhang et al. (37)	US	Cross sectional	6,157 (5798)	OBS	24-h food recalls	T3 VS. T1	MetS	0.25 (0.12, 0.51)	Age, sex, race/ethnicity, educational attainment, marital status, family income-to-poverty ratio, health insurance coverage, and total energy intake	16
			3,216 (3027)					Female 0.11 (0.05, 0.24)		
			2,941 (2771)					Male 0.59 (0.22, 1.62)		
			6,157 (NA)				Abdominal adiposity	0.41 (0.30, 0.57)		
			3,216 (NA)					Female 0.39 (0.24, 0.62)		
			2,941 (NA)					Male 0.46 (0.29, 0.72)		
			6,157 (NA)				Low HDL-cholesterol	0.68 (0.51, 0.90)		
			3,216 (NA)					Female 0.57 (0.39, 0.84)		
			2,941 (NA)					Male 0.81 (0.56, 1.17)		
			6,157 (NA)				High serum triglyceride	0.71 (0.53, 0.92)		
			3,216 (NA)					Female 0.50 (0.32, 0.78)		
			2,941 (NA)					Male 0.83 (0.52, 1.33)		
			6,157 (NA)				Abnormal glucose homeostasis	0.61 (0.45, 0.81)		
			3,216 (NA)					Female 0.50 (0.35, 0.71)		
			2,941 (NA)					Male 0.74 (0.47, 1.16)		
			6,157 (NA)				Elevated blood pressure	0.53 (0.40, 0.69)		
			3,216 (NA)					Female 0.52 (0.37, 0.74)		
			2,941 (NA)					Male 0.62 (0.41, 0.93)		

FFQ, Food Frequency Questionnaire; MD, Mediterranean diet; DDS, dietary diversity score; MetS, metabolic syndrome; VD, vegetarian diet; HEI, healthy eating index; AHEI, alternative healthy eating index; DASH, dietary approaches to stop hypertension; DII, dietary inflammatory index; PDI, plant-based diet index; hPDI, healthful plant-based diet index; uPDI, unhealthful plant-based diet Index; PBF, healthy plant-based food; OBS, oxidative balance score; MIND, Mediterranean-DASH Intervention for neurodegenerative delay deit; DQI, diet quality index.

### 3.3 Methodological quality assessment and certainty of evidence

**Figure 2:**
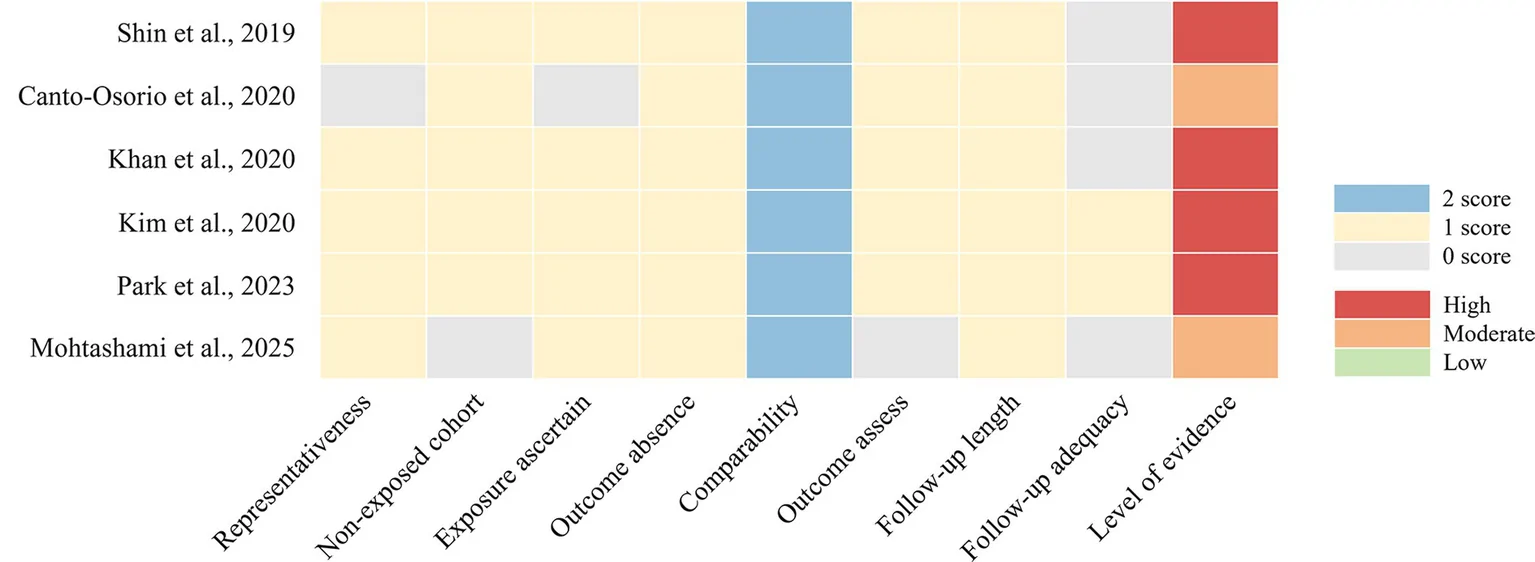
Quality appraisal of case–control and cohort studies via NOS.

To evaluate the study quality, the JBI was employed for 34 studies, and the NOS for 6 studies. For studies assessed using the JBI, 29 studies fulfilled all criteria, while 5 studies failed to satisfy several criteria. The main shortcomings were unclear descriptions of participants’ inclusion and exclusion criteria or inappropriately defined eligibility criteria. Based on the evaluation of NOS, the median score was 8, with scores ranging from 6 to 9. Two studies were rated as moderate quality and 4 as high quality. Details are presented in Figure 2 and Supplementary Table 4.

This study yielded 28 meta-analytic estimates of the association between 6 prior dietary patterns and MetS. After credibility assessment using GRADE, 11 (39.29%) were graded as low certainty, and 17 (60.71%) as very low certainty. The primary reason for downgrading was inconsistency. Details are presented in Supplementary Table 5.

### 3.4 Association of *a priori* dietary patterns with MetS

**Figure 3:**
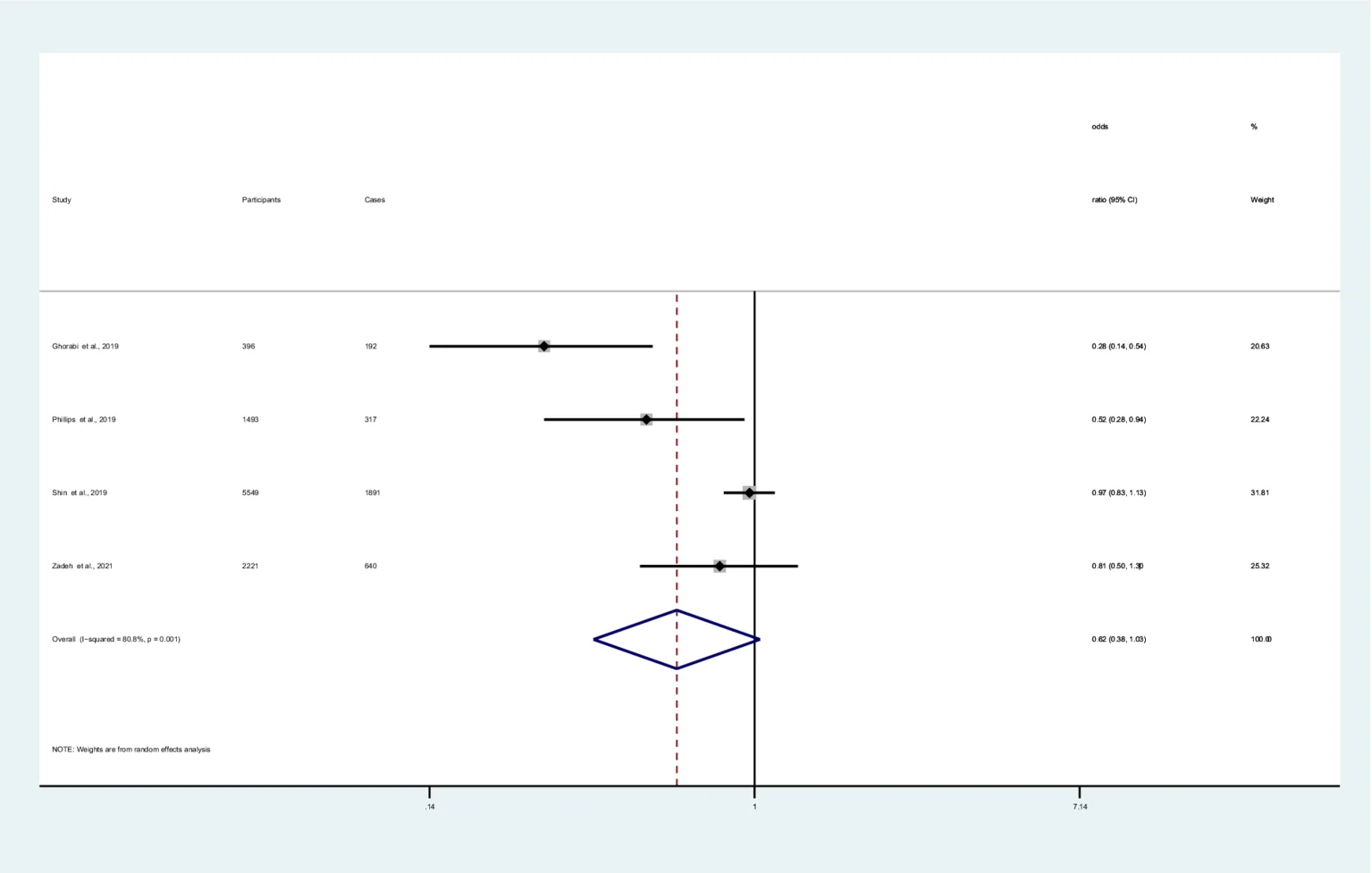
Forest plot for the association between DASH and MetS. DASH, dietary approaches to stop hypertension; MetS, metabolic syndrome.

**Figure 4:**
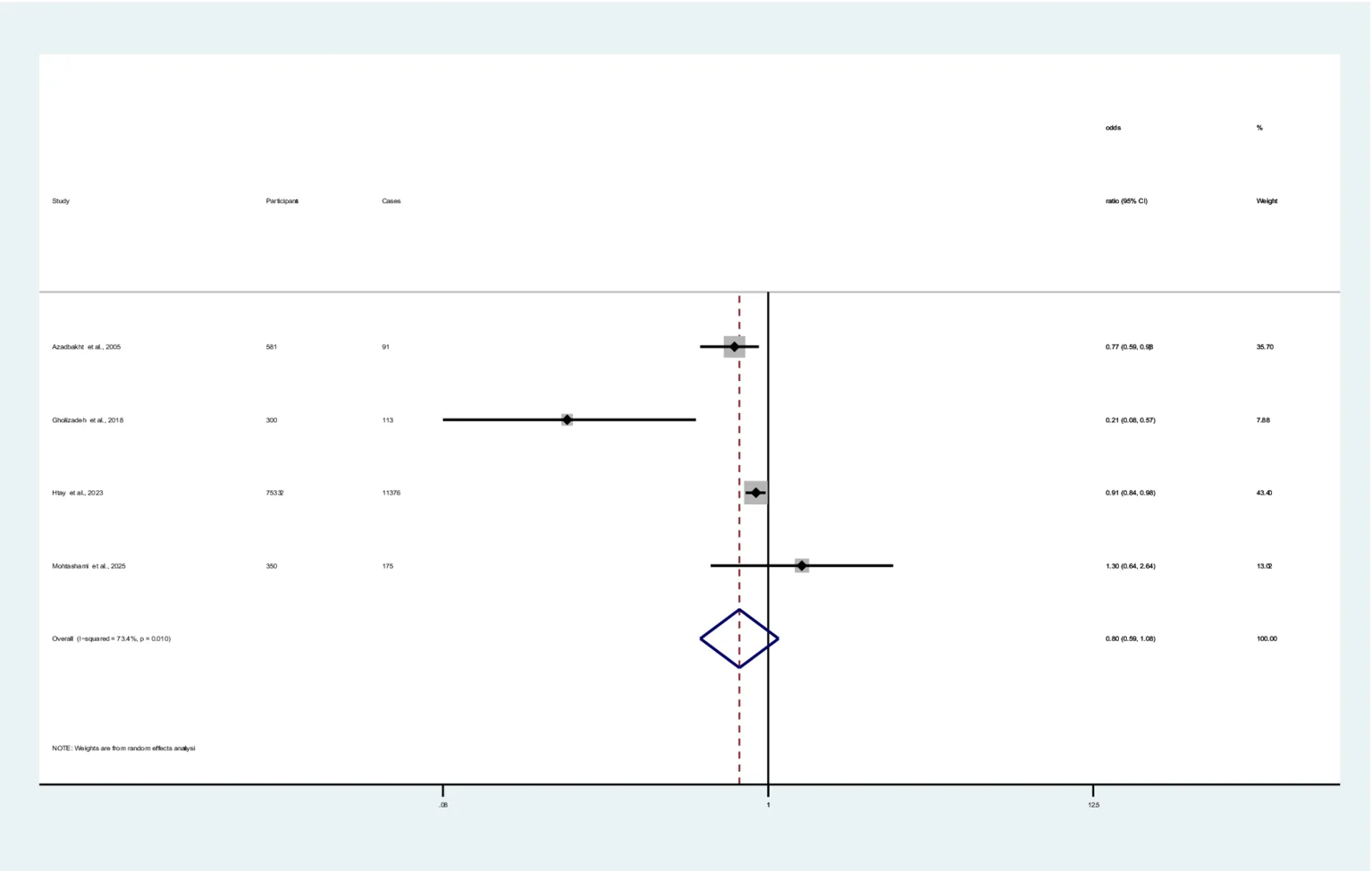
Forest plot for the association between DDS and MetS. DDS, dietary diversity score; MetS, metabolic syndrome.

**Figure 5:**
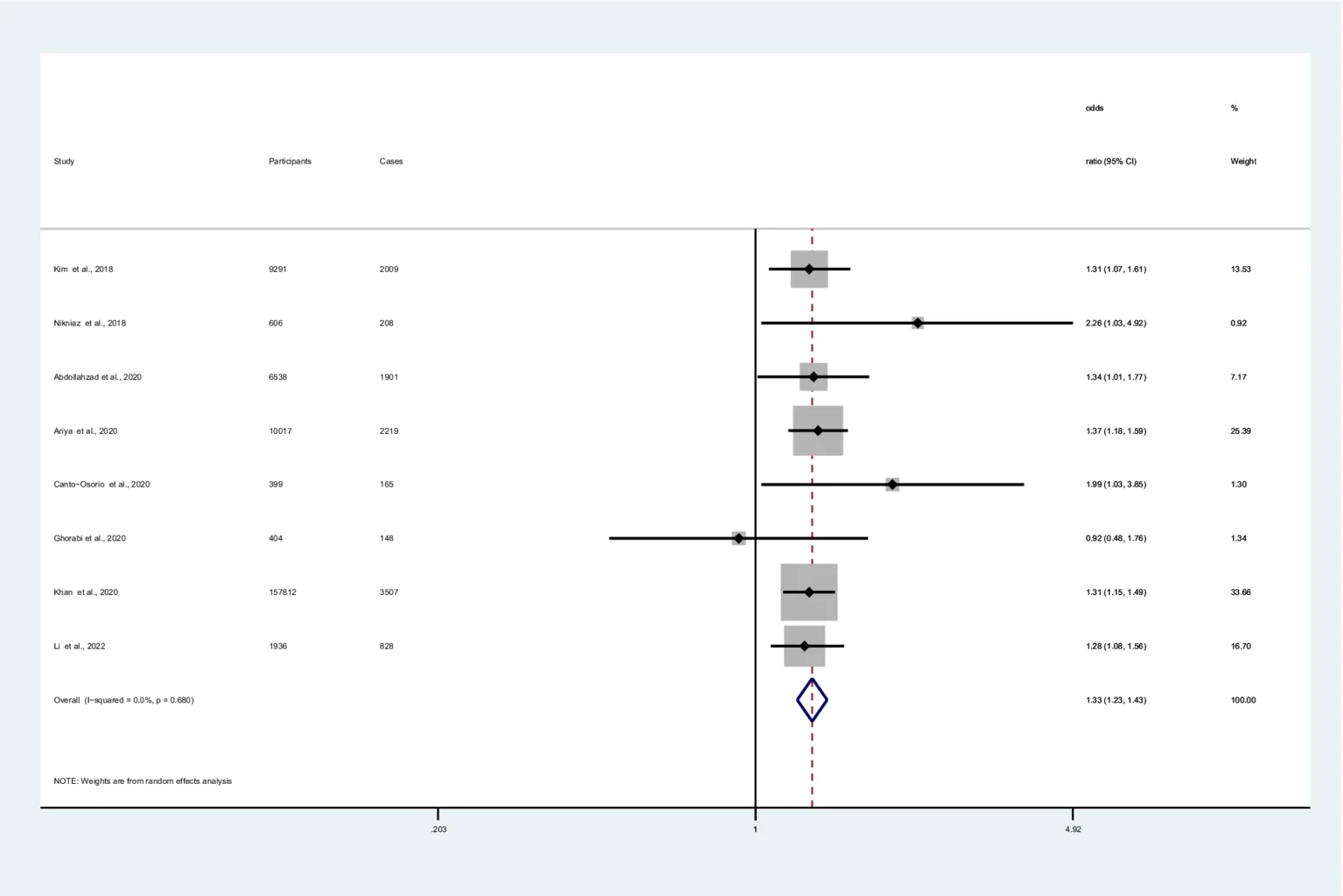
Forest plot for the association between DII and MetS. DII, dietary inflammatory index; MetS, metabolic syndrome.

**Figure 6:**
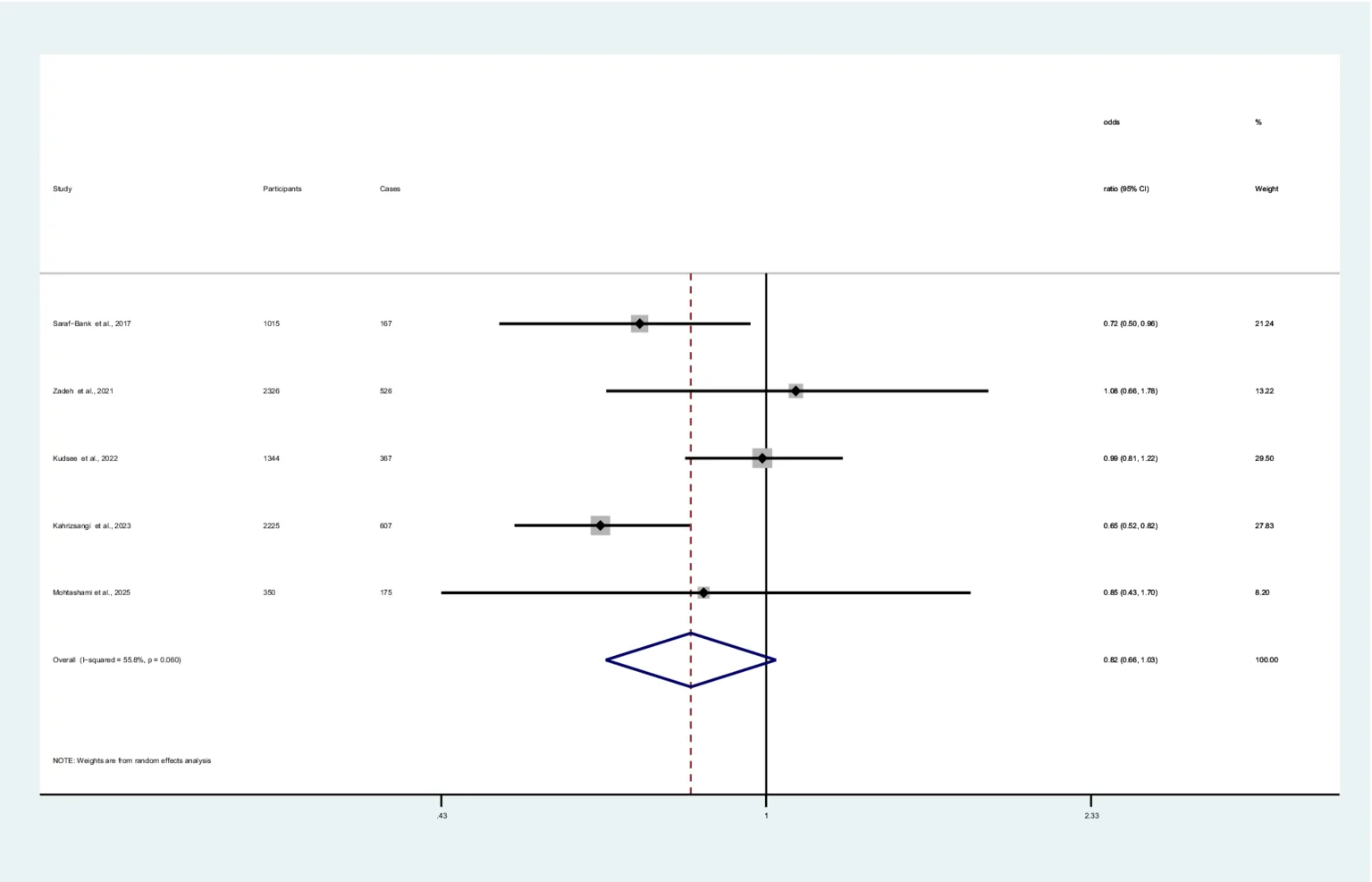
Forest plot for the association between HEI and MetS. HEI, Healthy Eating Index; MetS, metabolic syndrome.

**Figure 7:**
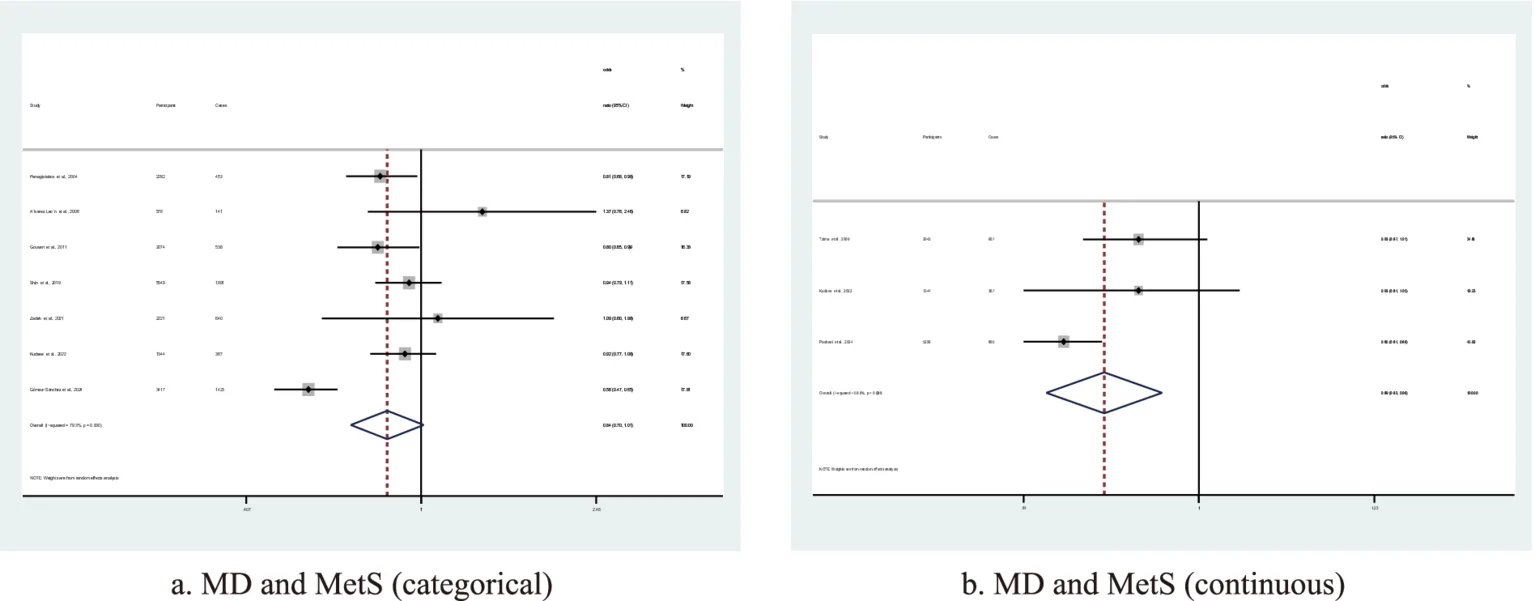
Forest plots for the association between MD and MetS. **(a)** MD and MetS (categorical); **(b)** MD and MetS (continuous). MD, Mediterranean diet; MetS, metabolic syndrome.

**Figure 8:**
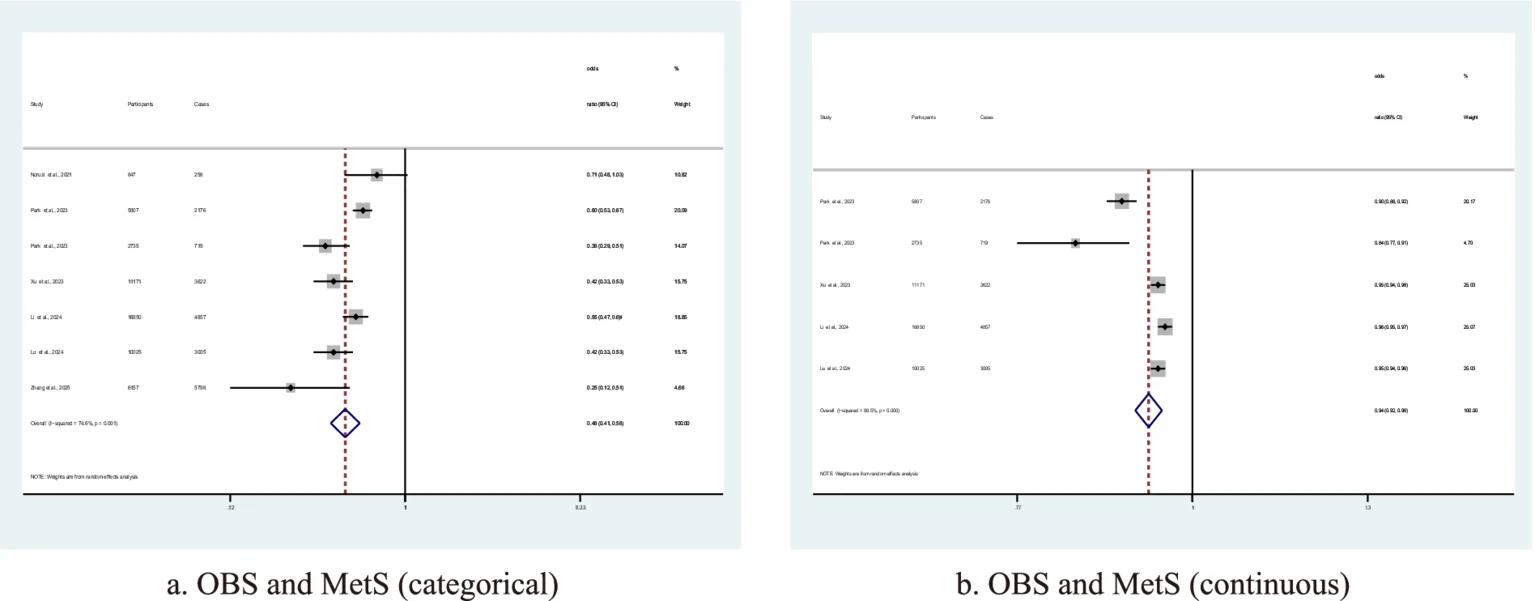
Forest plots for the association between OBS and MetS. **(a)** OBS and MetS (categorical) **(b)** OBS and MetS (continuous). OBS, Oxidative Balance Score; MetS, metabolic syndrome.

Strong compliance with the DASH diet was not significantly associated with MetS (OR [95% CI]: 0.62 [0.38–1.03], *p* > 0.05; *I*^2^ = 80.8%) (Figure 3). Similarly, no pronounced association was noted for DDS (0.80 [0.59–1.08], *p* > 0.05; *I*^2^ = 73.4%) (Figure 4). A marked link was found between the high DII group and a higher prevalence of MetS (1.33 [1.23–1.43], *p* < 0.001; *I*^2^ = 0.0%) (Figure 5). Greater compliance with HEI demonstrated no significant association with MetS (0.82 [0.66–1.03], *p* > 0.05; *I*^2^ = 55.8%) (Figure 6). For MD, the high adherence group showed no significant association with MetS relative to the low adherence group (0.84 [0.70–1.01], *p* > 0.05; *I*^2^ = 79.3%) (Figure 7a). However, each 1-point elevation in the score of MD corresponded to a significantly lower prevalence of MetS (0.89 [0.83–0.96], *p* = 0.001; *I*^2^ = 58.8%) (Figure 7b). For OBS, the high score group exhibited a significantly lower prevalence of MetS than the low score group (0.48 [0.41–0.58], *p* < 0.001; *I*^2^ = 74.6%) (Figure 8a). Furthermore, a 1-point increment in OBS corresponded to a significantly reduced prevalence of MetS (0.94 [0.92–0.96], *p* < 0.001; *I*^2^ = 88.5%) (Figure 8b).

### 3.5 Association of *a priori* dietary patterns with components of MetS

All results regarding the association between *a priori* dietary patterns and components of MetS are presented in Supplementary Table 6.

The high DII group was significantly linked to a higher prevalence of abdominal adiposity (OR [95% CI]: 1.49 [1.12–2.00], *p* < 0.01; *I*^2^ = 89.4%). Similarly, a significant association was found for low HDL-cholesterol (1.24 [1.00–1.52], *p* < 0.05; *I*^2^ = 84.6%). Significant associations were also noted for abnormal glucose homeostasis (1.19 [1.12–1.26], *p* < 0.001) and elevated blood pressure (1.25 [1.18–1.32], *p* < 0.001); neither analysis exhibited heterogeneity (*I*^2^ = 0.0%). No significant association was detected for high serum triglyceride (1.16 [0.95–1.42], *p* > 0.05; *I*^2^ = 86.1%).

Close compliance with HEI was significantly related to a reduced prevalence of high serum triglyceride (0.74 [0.61–0.88], *p* = 0.001) and abnormal glucose homeostasis (0.82 [0.68–0.99], *p* < 0.05), with no heterogeneity observed in both analyses (*I*^2^ = 0.0%). No significant associations were detected for abdominal adiposity (0.85 [0.70–1.03], *p* > 0.05), low HDL-cholesterol (0.91 [0.70–1.17], *p* > 0.05), and elevated blood pressure (0.79 [0.52–1.19], *p* > 0.05). No heterogeneity was noted for abdominal adiposity (*I*^2^ = 0.0%), whereas moderate heterogeneity was found for low HDL-cholesterol (*I*^2^ = 71.6%) and elevated blood pressure (*I*^2^ = 71.7%).

High adherence to OBS exhibited a marked protective effect on all five components of MetS (all *p* < 0.001). The strongest association was noted for abdominal adiposity (0.40 [0.27–0.61]; *I*^2^ = 94.7%). Significant protective associations were also noted for elevated blood pressure (0.61 [0.54–0.70]; *I*^2^ = 12.4%), high serum triglycerides (0.63 [0.55–0.72]; *I*^2^ = 41.5%), and abnormal glucose homeostasis (0.70 [0.63–0.78]; *I*^2^ = 0.0%). Low HDL-cholesterol showed a similarly significant association (0.62 [0.52–0.74]), albeit with moderate heterogeneity (*I*^2^ = 61.2%). With rising OBS, the prevalence of all components of MetS was significantly diminished (all *p* < 0.001): abdominal adiposity (0.94 [0.92–0.95]; *I*^2^ = 84.6%), elevated blood pressure (0.97 [0.96–0.98]; *I*^2^ = 74.0%), low HDL-cholesterol (0.96 [0.96–0.97]; *I*^2^ = 22.2%), high serum triglyceride (0.97 [0.95–0.98]; *I*^2^ = 80.6%), and abnormal glucose homeostasis (0.98 [0.97–0.98]; *I*^2^ = 11.6%).

Fewer than three eligible studies examined the associations of DDS, MD and DASH with individual components of MetS. Considering that pooled effect estimates tend to be unreliable with fewer than three included studies, quantitative pooled analyses were not conducted for these three dietary patterns.

### 3.6 Subgroup analysis

#### 3.6.1 Stratification by sex

Sex-stratified subgroup analyses were implemented for both MetS and its components when sufficient data were available. Regarding MetS, the findings of sex-stratified analyses for DII, HEI, MD, and OBS were consistent with the main analysis (Supplementary Table 7). For DII, both male and female subgroups exhibited significantly higher prevalence of MetS. For HEI, subgroup analysis was only carried out in females due to insufficient data in males, revealing no marked association with MetS. For MD, neither male nor female subgroups demonstrated a pronounced association with MetS. Greater compliance with OBS was related to a significantly reduced prevalence of MetS in both male and female subgroups. Moreover, a 1-point increment in OBS was correlated with a reduced prevalence of MetS in both males and females.

Regarding individual components of MetS, sex-stratified analyses were only feasible for DII (all five components) and HEI (only abnormal glucose homeostasis in females), and the results also corroborated the main findings (Supplementary Table 7). For abdominal adiposity, the high DII group was associated with a significantly higher prevalence in males and females, with reduced heterogeneity after sex-stratification. For low HDL-cholesterol, no marked association was found in males or females, with a consistent effect direction. For high serum triglycerides, elevated DII was significantly linked to a higher prevalence in males and females. Stratification by sex substantially reduced the high heterogeneity noted in the overall population. For elevated blood pressure, the high DII group was associated with a significantly higher prevalence in males and females. For abnormal glucose homeostasis, no marked association was observed for the high DII group in either males or females, while no heterogeneity was detected in the overall analysis; substantial heterogeneity was noted in both sex subgroups. The subgroup analysis of HEI was limited to females due to insufficient data on males and also showed no significant association, with a consistent trend with the main analysis.

#### 3.6.2 Stratification by study design

Subgroup analyses by study design were conducted to explore the associations of *a priori* dietary patterns with MetS and its components. Due to the limited number of cohort and case–control studies, stratified analyses were only performed among cross-sectional studies (Supplementary Table 8). After stratification, DII showed a statistically significant association with MetS. For the DASH diet, the association became significant, and heterogeneity decreased from high to moderate. No significant association was observed for MD, DDS, or HEI. Among all dietary patterns, only OBS was assessed in both categorical and continuous forms. In categorical analysis, OBS was significantly associated with MetS. Higher OBS was related to a lower prevalence of MetS, and heterogeneity declined from high to moderate after stratification.

Due to the limited number of eligible studies, stratified analyses for individual components of MetS were only performed for DII (Supplementary Table 8). Elevated DII showed no significant association with abdominal adiposity. No significant association was observed between higher DII and low HDL-cholesterol. Higher DII was not significantly associated with high serum triglycerides. Elevated DII was significantly associated with abnormal glucose homeostasis. A significant association existed between higher DII and elevated blood pressure.

#### 3.6.3 Stratification by continent

Subgroup analyses by continent were performed for the association between *a priori* dietary patterns and MetS. Due to the limited number of included studies, stratified analyses were only feasible for certain continents (Supplementary Table 9). For DII, a significant association with MetS was observed in the Asian subgroup. The findings remained stable across multiple stratified analyses. For the DASH diet, no significant association was observed in the Asian subgroup. For HEI, a significant association emerged in the Asian subgroup after stratification, with heterogeneity decreasing from moderate to low. For MD, no significant association was detected in the European subgroup. For categorical OBS, significant associations were found in both the Asian and North American subgroups, with heterogeneity declining from high to moderate in the latter. For continuous OBS, a significant association persisted in the North American subgroup after stratification, with heterogeneity decreasing substantially from high to low.

Subgroup analyses by continent were also conducted for individual components of MetS. Due to the limited number of included studies, stratified analyses were only feasible for certain continents and were limited to categorical dietary pattern variables (Supplementary Table 9). For abdominal adiposity, a significant association was observed in the Asian subgroup of DII. A significant association was also found in the North American subgroup of OBS. Heterogeneity remained high. For low HDL-cholesterol, no significant association was observed in the Asian subgroup of DII. A significant association was found in the North American subgroup of OBS. This association was significant in the primary analysis. Heterogeneity decreased from moderate to zero after stratification by North America. For high serum triglycerides, no significant association was observed in the Asian subgroup of DII. A significant association was found in the North American subgroup of OBS. For abnormal glucose homeostasis, a significant association was observed in the Asian subgroup of DII. A significant association was also found in the North American subgroup of OBS. For elevated blood pressure, a significant association was observed in the Asian subgroup of DII (the only subgroup with available data). The findings remained stable across multiple stratified analyses.

### 3.7 Sensitivity analysis

Leave-one-out sensitivity analysis was implemented for all 28 primary analyses. The results of 19 analyses remained statistically consistent after sequentially omitting individual studies. Nevertheless, statistical significance was altered in 9 analyses (1 for DII, 1 for DASH, 1 for DDS, and 6 for HEI), suggesting these results were sensitive (Supplementary Figures 1–7). Notably, HEI-related findings were relatively more sensitive, which may be attributed to the inclusion of different versions of HEI and the small number of relevant studies. Thus, these results should be interpreted cautiously.

## 4 Discussion

The present meta-analysis found that the high DII group was associated with a higher prevalence of MetS and its components. High adherence to OBS was significantly related to a reduced prevalence of MetS and all its components. A one-unit increment in the MD score was associated with a lower prevalence of MetS in continuous analyses, while no significant association was observed between groups with high and low adherence to the MD. Nonetheless, DASH, DDS, and HEI demonstrated no significant associations with MetS. These findings suggest that *a priori* dietary patterns may have potential roles in the prevention and management of MetS.

A significant positive association of DII with MetS was noted, aligning with previous epidemiological studies (50, 54). A large Korean cohort study of 150,000 adults followed for 7.5 years reports that a more pro-inflammatory diet is related to a heightened risk of incident MetS and its components, with the most prominent association seen for HDL-cholesterol (54). Similarly, a cross-sectional study in older Chinese individuals shows that elevated DII is correlated with greater odds of prevalent MetS and most of its components. Body mass index (BMI)-stratified analyses indicate that this association remains stable across all BMI categories, suggesting that the link between DII and MetS is independent of adiposity levels (50). DII is a validated *a priori* index that captures the inflammatory potential of the overall diet. Greater scores correspond to a more pro-inflammatory dietary profile (72). Sustained low-grade inflammation is considered a pivotal driver in the occurrence of MetS (73). Prolonged inflammatory signals may affect insulin signaling pathways and induce insulin resistance, thereby causing disturbances in lipid metabolism (74, 75).

In the current research, OBS was determined as a marked protective factor for MetS and all its components. High adherence to the OBS was associated with a lower prevalence of MetS and its components. These findings align with prior research (44, 51). In a prospective Korean cohort of nearly 6,000 middle-aged and older adults followed for almost 14 years, strong compliance with OBS was related to a diminished risk of MetS, with pronounced protective effects in both men and women (44). This emphasizes the significance of adequate intake of antioxidants, regular physical activity, and smoking cessation for preventing MetS. Besides, OBS has also been identified as a significant protective factor for MetS in a large US cross-sectional study of more than 10,000 adults (51). Pronounced protective associations with all five components of MetS are noted for OBS, reinforcing the beneficial role of healthy dietary patterns in lowering the prevalence of metabolic diseases (49). As a well-recognized healthy dietary pattern, OBS is marked by high consumption of whole grains, fruits, vegetables, and legumes, and low consumption of ultra-processed foods, added sugars, and saturated fats (76). Its protective effects may be attributed to the combined actions of multiple nutrients. Dietary fiber and antioxidants can reduce oxidative stress and persistent inflammation, improve insulin sensitivity, and maintain normal lipid metabolism (77). Additionally, whole grains and legumes help maintain a healthy gut microbiota, decrease visceral fat accumulation, and support normal blood pressure (78). In the analyses of OBS and MetS as well as its components, most results showed substantial heterogeneity. Several factors may have contributed to this heterogeneity. The dietary assessment tools used in the included studies were inconsistent. Some studies used food frequency questionnaires (FFQs), whereas others used 24-h dietary recalls. Study samples came from different countries, and both cross-sectional and cohort designs were adopted. In addition, the baseline characteristics of study populations varied. Some studies enrolled all adults, while others focused only on middle-aged and older adults. These methodological, geographical and population differences may have led to the between-study heterogeneity across analyses. Notably, subgroup analyses stratified by continent further supported geographical variation as an important source of heterogeneity. Heterogeneity was markedly reduced among North American studies.

MD is a traditional dietary pattern characterized by high consumption of whole grains, vegetables, fruits, legumes, olive oil, and fish, which is associated with favorable metabolic health (79). In the present research, no substantial difference was discovered between participants with high and low adherence to MD. Conversely, a higher score of MD exhibited a pronounced inverse association with MetS and its components. Such divergent findings between categorical and continuous analyses can be partly attributed to differences in statistical power and data utilization. Continuous MD scores retain adherence information from all participants, leading to higher statistical power. By comparison, categorical analyses only contrast participants in two extreme groups and discard information from roughly half of individuals with moderate adherence. Moreover, different cut-off values used to define high and low MD adherence across studies may introduce additional heterogeneity. The categorical meta-analysis showed substantial heterogeneity, alongside wide CIs spanning the null value, which impedes the identification of a meaningful association. Existing research on the association of MD with MetS has reported inconsistent findings (18, 19, 43), even though most investigations indicate that MD has a protective effect. The current findings are consistent with most prior studies (19, 43), showing progressively lower prevalence of MetS with escalating scores of MD. One cross-sectional study, conducted in Spain, includes nearly 3,500 adult participants. It reveals a significant protective effect of MD on MetS and its five components (19). Another cross-sectional study from Iran enrolls more than 5,000 adults and reports that each 1-unit increment in the score of MD corresponds to a 14.5% lower prevalence of MetS (43). Conversely, a cross-sectional study of over 2,000 adults (18) in Iran reveals no significant association of MD with the prevalence of MetS. Nevertheless, the same study reports that MD effectively reduces fasting blood glucose and abdominal obesity in female participants, thereby conferring a protective effect on specific components of MetS. These inconsistent findings may stem from variations in study populations, regional dietary structures, eating habits, and the scoring standards of MD. Notably, substantial heterogeneity existed in analyses of MD. Several potential reasons account for this heterogeneity. First, baseline consumption of olive oil and fish differs across regions, leading to inconsistent protective effects of MD on MetS (80). Second, inconsistent dietary survey tools were used, including FFQs and specialized nutrition questionnaires. Third, samples were collected from six countries with both cross-sectional and cohort designs. Fourth, diverse MD scoring systems were applied. Moderate alcohol intake was scored in some indices, while alcohol-related items were excluded in others. All the above differences may jointly lead to the between-study heterogeneity.

No significant association with MetS was noted for DASH, DDS, or HEI. DASH was originally developed to lower blood pressure. Its cardioprotective and metabolic benefits are well established in clinical trials (81, 82). Notably, sensitivity analyses indicated that the findings of DASH were sensitive to the study by Shin et al. (2019) (23). Omitting that study yielded a statistically significant protective effect. DDS reflects the variety and balance of food groups consumed, and higher dietary diversity is theoretically linked to adequate intake of nutrients, reduced inflammation, and favorable metabolic profiles (83). Despite evidence linking low dietary diversity to adverse metabolic outcomes (84), the present research revealed no significant association of DDS with MetS. This result is likely due to the very small number of eligible studies. HEI is a scoring system that evaluates adherence to official dietary guidelines and reflects overall dietary quality (85). The current meta-analysis detected a protective effect of HEI only on high serum triglycerides and abnormal glucose homeostasis, but not for overall MetS. Sensitivity analyses indicated that HEI-related findings were relatively unstable, which may be attributed to different versions of HEI (AHEI, HEI-2010, HEI-2015) employed across studies and the small number of available datasets. These findings should therefore be interpreted with caution. Between-study heterogeneity was also observed in the pooled analyses of DASH, DDS and HEI. Such inconsistency can be partly attributed to different dietary assessment tools, diverse study designs, as well as varied age distributions and national backgrounds of participants, which also explains the unstable results detected in leave-one-out sensitivity analyses. Subgroup analyses further supported this speculation. HEI showed a significant association within the Asian subgroup accompanied by reduced heterogeneity. For DASH diet, significant association was identified in cross-sectional subgroup, with heterogeneity declining from high to moderate.

The current findings are broadly consistent with previous meta-analyses on dietary patterns and metabolic health. Prior quantitative syntheses have shown that DII (86) and MD (87) are associated with metabolic disorders. No relevant meta-analyses concerning OBS and MetS were retrieved in our literature search, and available evidence is restricted to clinical studies. Most previous meta-analyses reported a protective association between the DASH diet and MetS (88–90). However, no significant pooled association was detected in our main analysis. This finding should be interpreted with caution, as it may reflect limited statistical power, differences in dietary assessment methods, and population heterogeneity. For HEI, no relevant meta-analyses on its association with MetS were identified during the literature search. Nevertheless, existing meta-analyses have reported associations between HEI and other metabolic diseases, such as T2DM (91) and metabolic dysfunction-associated steatotic liver disease (92). In this analysis, HEI was not significantly associated with MetS, whereas significant associations were observed for triglycerides and blood glucose levels. These findings are consistent with those reported in previous meta-analyses. For DDS, the present results are broadly consistent with two published meta-analyses (93, 94). One study reported no significant association between DDS and MetS (94), while another found no association between DDS and BMI (93). Two factors may explain the absence of significant associations. First, differences in dietary assessment protocols and study populations exist across studies. Second, DDS only counts food categories without distinguishing their health impacts or pro-inflammatory properties. A diet with high diversity may still include substantial amounts of refined carbohydrates, added sugars, and processed meats, which may promote inflammation, oxidative stress, and metabolic abnormalities.

Meta-analyses of a posteriori dietary patterns provide evidence that is consistent with our findings. Data-driven approaches generally identify broadly healthy and unhealthy dietary patterns. Healthy dietary patterns are typically characterized by higher intakes of vegetables, fruits, whole grains, legumes, nuts, fish, and other foods rich in antioxidant and anti-inflammatory nutrients. In contrast, unhealthy dietary patterns often include higher intakes of refined grains, added sugars, sugar-sweetened beverages, processed meats, and energy-dense foods. These dietary characteristics overlap with the dietary indices evaluated in the present study. A lower DII indicates a lower overall inflammatory potential of the diet. It may reflect higher consumption of anti-inflammatory foods and lower consumption of pro-inflammatory foods. A higher OBS indicates greater intake of antioxidant components and lower exposure to pro-oxidant dietary factors. Previous meta-analyses of a posteriori dietary patterns have shown that healthy dietary patterns rich in anti-inflammatory and antioxidant foods are generally associated with a more favorable metabolic profile (95–97). This evidence is in line with our findings. Taken together, evidence from both *a priori* and a posteriori dietary pattern studies suggests that dietary patterns rich in anti-inflammatory and antioxidant components may be associated with a lower prevalence of MetS and its individual components.

Accumulating evidence indicates that chronic low-grade inflammation and oxidative stress are involved in the development and progression of MetS (98). Dietary patterns may influence these processes by modifying inflammatory mediators (99), antioxidant capacity (100), and gut microbiota composition (101). Thus, the observed associations of DII and OBS with MetS are biologically plausible. However, most of the included studies were observational, and many were cross-sectional. Therefore, these findings should be interpreted as associations rather than causal effects. Further prospective cohort studies and well-designed intervention trials are needed to verify the potential causal relationships between these dietary patterns and MetS.

The present research has its strengths. First, this is the first comprehensive meta-analysis to synthesize the associations of six widely investigated *a priori* dietary patterns with MetS and all its five components, thereby providing a holistic understanding of these associations. Second, a multidimensional analytical approach was adopted, including categorical, continuous, and sex-stratified analyses, alongside leave-one-out sensitivity analyses, which fully explored the possible associations and between-study heterogeneity. Third, the eligible studies were conducted across 11 countries spanning multiple continents with a large aggregate sample size. This extends the generalizability of our findings and provides cross-population evidence for the associations of *a priori* dietary patterns with MetS.

Nevertheless, several limitations should be considered. First, most included studies were cross-sectional, which limits causal inference and may introduce reverse causality, as participants with MetS may have changed their diet after diagnosis. We could not convert effect estimates from various study designs into consistent RRs due to insufficient reported data. Direct pooling of these effect sizes on the logarithmic scale may overestimate the magnitude of associations. In addition, heterogeneity may also arise from differences in study design, despite the use of a random-effects model. However, subgroup analyses were conducted based on study design, region, and sex to better explain the sources of heterogeneity. Second, dietary assessment tools, mainly FFQs and 24-h dietary recalls, are prone to measurement error and recall bias, which may cause exposure misclassification and usually attenuate the true associations toward the null. Third, grey literature databases were not systematically searched, which may lead to publication bias and overestimate the association between *a priori* dietary patterns and MetS. Furthermore, because of the small number of eligible studies, the evaluation of publication bias using Egger’s test or funnel plot was not conducted. Fourth, the number of eligible studies was small for some dietary indices and components of MetS, which reduced statistical power and made some estimates less stable, especially for HEI-related findings. Finally, the restriction to studies using the NCEP-ATP III definition improved diagnostic consistency but may limit generalizability.

## 5 Conclusion

The present meta-analysis evaluated the associations of six *a priori* dietary patterns with the prevalence of MetS and its components. High adherence to DII was associated with elevated prevalence of MetS, whereas high OBS and MD scores were related to reduced prevalence of MetS. These findings suggest a potential role of dietary patterns in supporting metabolic health. Diets rich in anti-inflammatory and antioxidant components and Mediterranean-style eating patterns may offer potential implications for the prevention and management of MetS. However, most included studies are cross-sectional, and thus the causal relationship between dietary patterns and MetS is difficult to establish. Further well-designed prospective cohorts and randomized trials are needed to clarify whether higher adherence to *a priori* dietary patterns can reduce the risk of MetS.

## Data Availability

The original contributions presented in the study are included in the article/Supplementary material, further inquiries can be directed to the corresponding author.
