# Supplementary material for: Association of priori dietary patterns with metabolic syndrome and its components: a systematic review and meta-analysis
Source: Front Nutr. 2026 Sep 4;13:1882985. doi: 10.3389/fnut.2026.1882985 (PMC13585732; doi:10.3389/fnut.2026.1882985)
Supplement: Supplementary file 1 [file Supplementary_file_1.DOCX]

**Supplementary materials**

**Supplementary Table 1** Search Strategies.

**Search strategy in PubMed, Embase and Web of Science from inception to March 20, 2025.**

| Bibliographic databases | Query | Number of Articles |
| --- | --- | --- |
| PubMed | #1: ((((((((((((((((((((Dietary Pattern[Title/Abstract]) OR (Mediterranean Diet[Title/Abstract])) OR (Dietary Approaches to Stop Hypertension[Title/Abstract])) OR (eating habit[Title/Abstract])) OR (eating pattern[Title/Abstract])) OR (food pattern[Title/Abstract])) OR (food quality[Title/Abstract])) OR (food habit[Title/Abstract])) OR (Healthy Eating Index[Title/Abstract])) OR (Alternative Healthy Eating Index[Title/Abstract])) OR (alternate Mediterranean Diet[Title/Abstract])) OR (Modified Mediterranean Diet[Title/Abstract])) OR (Dietary diversity score[Title/Abstract])) OR (Diet Quality Index-International[Title/Abstract])) OR (Oxidative balance score[Title/Abstract])) OR (Empirical dietary inflammatory pattern[Title/Abstract])) OR (carbohydrate quality index[Title/Abstract])) OR (dietary inflammatory index[Title/Abstract])) OR (Plant-based Diet[Title/Abstract])) OR (vegetarian[Title/Abstract])) OR (Chinese Healthy Eating Index[Title/Abstract])  #2: (Metabolic syndrome[Title/Abstract]) OR (MetS[Title/Abstract])  #3: ((((((((Observational[Title/Abstract]) OR (case control[Title/Abstract])) OR (Case-control[Title/Abstract])) OR (Cross sectional[Title/Abstract])) OR (Cross-sectional[Title/Abstract])) OR (Prevalence[Title/Abstract])) OR (Cohort[Title/Abstract])) OR (Prospective[Title/Abstract])) OR (retrospective[Title/Abstract])  #4: #1 AND #2 AND #3 | 738 |
| Embase | #1: 'dietary pattern':ab,ti OR 'mediterranean diet':ab,ti OR 'dietary approaches to stop hypertension':ab,ti OR 'eating habit':ab,ti OR 'eating pattern':ab,ti OR 'food pattern':ab,ti OR 'food quality':ab,ti OR 'food habit':ab,ti OR 'healthy eating index':ab,ti OR 'alternative healthy eating index':ab,ti OR 'alternate mediterranean diet':ab,ti OR 'modified mediterranean diet':ab,ti OR 'dietary diversity score':ab,ti OR 'diet quality index-international':ab,ti OR 'oxidative balance score':ab,ti OR 'empirical dietary inflammatory pattern':ab,ti OR 'carbohydrate quality index':ab,ti OR 'dietary inflammatory index':ab,ti OR 'plant-based diet':ab,ti OR vegetarian:ab,ti OR 'chinese healthy eating index':ab,ti  #2: 'metabolic syndrome':ab,ti OR mets:ab,ti  #3: observational:ab,ti OR 'case control':ab,ti OR 'cross sectional':ab,ti OR prevalence:ab,ti OR cohort:ab,ti OR prospective:ab,ti OR retrospective:ab,ti  #4: #1 AND #2 AND #3 | 885 |
| Web of Science | #1: ((((((((((((((((((((AB=(Dietary Pattern)) OR AB=(Mediterranean Diet)) OR AB=(Dietary Approaches to Stop Hypertension)) OR AB=(eating habit)) OR AB=(eating pattern)) OR AB=(food pattern)) OR AB=(food quality)) OR AB=(food habit)) OR AB=(Healthy Eating Index)) OR AB=(Alternative Healthy Eating Index)) OR AB=(alternate Mediterranean Diet)) OR AB=(Modified Mediterranean Diet)) OR AB=(Dietary diversity score)) OR AB=(Diet Quality Index-International)) OR AB=(Oxidative balance score)) OR AB=(Empirical dietary inflammatory pattern)) OR AB=(carbohydrate quality index)) OR AB=(dietary inflammatory index)) OR AB=(Plant-based Diet)) OR AB=(vegetarian)) OR AB=(Chinese Healthy Eating Index)  #2: (AB=(Metabolic syndrome)) OR AB=(MetS)  #3: ((((((((AB=(Observational)) OR AB=(case control)) OR AB=(Case-control)) OR AB=(Cross sectional)) OR AB=(Cross-sectional)) OR AB=(Prevalence)) OR AB=(Cohort)) OR AB=(Prospective)) OR AB=(retrospective)  #4: ((#1) AND #2) AND #3 | 1227 |

**Supplementary Table 2** List of the excluded records during the process of full-text review.

**Inconsistent diagnostic criteria (n=37)**

[1] Tortosa A, Bes-Rastrollo M, Sanchez-Villegas A, Basterra-Gortari FJ, Nuñez-Cordoba JM, Martinez-Gonzalez MA. Mediterranean diet inversely associated with the incidence of metabolic syndrome: the SUN prospective cohort. Diabetes Care.(2007) 30(11), 2957-2959. doi:10.2337/dc07-1231

[2] Gregory CO, McCullough ML, Ramirez-Zea M, Stein AD. Diet scores and cardio-metabolic risk factors among Guatemalan young adults. Br J Nutr.(2009) 101(12), 1805-1811. doi:10.1017/S000711450813584X

[3] Kesse-Guyot E, Ahluwalia N, Lassale C, Hercberg S, Fezeu L, Lairon D. Adherence to Mediterranean diet reduces the risk of metabolic syndrome: a 6-year prospective study. Nutr Metab Cardiovasc Dis.(2013) 23(7), 677-683. doi:10.1016/j.numecd.2012.02.005

[4] Chiang JK, Lin YL, Chen CL, Ouyang CM, Wu YT, Chi YC, et al. Reduced risk for metabolic syndrome and insulin resistance associated with ovo-lacto-vegetarian behavior in female Buddhists: a case-control study. PLoS One.(2013) 8(8), e71799. doi:10.1371/journal.pone.0071799

[5] Yang J, Farioli A, Korre M, Kales SN. Modified Mediterranean diet score and cardiovascular risk in a North American working population. PLoS One.(2014) 9(2), e87539. doi:10.1371/journal.pone.0087539

[6] Saneei P, Fallahi E, Barak F, Ghasemifard N, Keshteli AH, Yazdannik AR, et al. Adherence to the DASH diet and prevalence of the metabolic syndrome among Iranian women. Eur J Nutr.(2015) 54(3), 421-428. doi:10.1007/s00394-014-0723-y

[7] Grosso G, Stepaniak U, Micek A, Topor-Mądry R, Stefler D, Szafraniec K, et al. A Mediterranean-type diet is associated with better metabolic profile in urban Polish adults: Results from the HAPIEE study. Metabolism.(2015) 64(6), 738-746. doi:10.1016/j.metabol.2015.02.007

[8] Neufcourt L, Assmann KE, Fezeu LK, Touvier M, Graffouillère L, Shivappa N, et al. Prospective association between the dietary inflammatory index and metabolic syndrome: findings from the SU.VI.MAX study. Nutr Metab Cardiovasc Dis.(2015) 25(11), 988-996. doi:10.1016/j.numecd.2015.09.002

[9] Klosowiak JL, Park S, Smith KP, French ME, Focia PJ, Freymann DM, et al. Structural insights into Parkin substrate lysine targeting from minimal Miro substrates. Sci Rep.(2016) 6, 33019. doi:10.1038/srep33019

[10] Sokol A, Wirth MD, Manczuk M, Shivappa N, Zatonska K, Hurley TG, et al. Association between the dietary inflammatory index, waist-to-hip ratio and metabolic syndrome. Nutr Res.(2016) 36(11), 1298-1303. doi:10.1016/j.nutres.2016.04.004

[11] Lavigne-Robichaud M, Moubarac JC, Lantagne-Lopez S, Johnson-Down L, Batal M, Laouan Sidi EA, et al. Diet quality indices in relation to metabolic syndrome in an Indigenous Cree (Eeyouch) population in northern Québec, Canada. Public Health Nutr.(2018) 21(1), 172-180. doi:10.1017/S136898001700115X

[12] Kim Y, Je Y. A modified Mediterranean diet score is inversely associated with metabolic syndrome in Korean adults. Eur J Clin Nutr.(2018) 72(12), 1682-1689. doi:10.1038/s41430-018-0156-4

[13] Misra R, Balagopal P, Raj S, Patel TG. Vegetarian Diet and Cardiometabolic Risk among Asian Indians in the United States. J Diabetes Res.(2018) 2018, 1675369. doi:10.1155/2018/1675369

[14] Navarro JCA, Antoniazzi L, Oki AM, Bonfim MC, Hong V, Bortolotto LA, et al. Prevalence of Metabolic Syndrome and Framingham Risk Score in Apparently Healthy Vegetarian and Omnivorous Men. Arq Bras Cardiol.(2018) 110(5), 430-437. doi:10.5935/abc.20180073

[15] Ren Z, Zhao A, Wang Y, Meng L, Szeto IM, Li T, et al. Association between Dietary Inflammatory Index, C-Reactive Protein and Metabolic Syndrome: A Cross-Sectional Study. Nutrients.(2018) 10(7), 831. doi:10.3390/nu10070831

[16] Joyce BT, Wu D, Hou L, Dai Q, Castaneda SF, Gallo LC, et al. DASH diet and prevalent metabolic syndrome in the Hispanic Community Health Study/Study of Latinos. Prev Med Rep.(2019) 15, 100950. doi:10.1016/j.pmedr.2019.100950

[17] Kenđel Jovanović G, Pavičić Žeželj S, Klobučar Majanović S, Mrakovcic-Sutic I, Šutić I. Metabolic syndrome and its association with the Dietary Inflammatory Index (DII)® in a Croatian working population. J Hum Nutr Diet.(2020) 33(1), 128-137. doi:10.1111/jhn.12695

[18] Ramón-Arbués E, Martínez-Abadía B, Granada-López JM, Echániz-Serrano E, Huércanos-Esparza I, Antón-Solanas I. Association between adherence to the Mediterranean diet and the prevalence of cardiovascular risk factors. Rev Lat Am Enfermagem.(2020) 28, e3295. doi:10.1590/1518-8345.3904.3295

[19] Xiao X, Qin Z, Lv X, Dai Y, Ciren Z, Yangla Y, et al. Dietary patterns and cardiometabolic risks in diverse less-developed ethnic minority regions: results from the China Multi-Ethnic Cohort (CMEC) Study. Lancet Reg Health West Pac.(2021) 15, 100252. doi:10.1016/j.lanwpc.2021.100252

[20] Pang T, Alman AC, Gray HL, Basu A, Shi L, Snell-Bergeon JK. Empirical dietary inflammatory pattern and metabolic syndrome: prospective association in participants with and without type 1 diabetes mellitus in the coronary artery calcification in type 1 diabetes (CACTI) study. Nutr Res.(2021) 94, 1-9. doi:10.1016/j.nutres.2021.08.001

[21] Abdurahman A, Bule M, Fallahyekt M, Abshirini M, Azadbakht L, Qorbani M, et al. Association of Diet Quality and Food Insecurity with Metabolic Syndrome in Obese Adults. Int J Prev Med.(2021) 12, 138. doi:10.4103/ijpvm.IJPVM_191_19

[22] Cui N, Ouyang Y, Li Y, Yang Y, Liu S, Li J, et al. Better adherence to the Chinese Healthy Eating Index is associated with a lower prevalence of metabolic syndrome and its components. Nutr Res.(2022) 104, 20-28. doi:10.1016/j.nutres.2022.02.008

[23] Cubas-Basterrechea G, Elío I, Alonso G, Otero L, Gutiérrez-Bardeci L, Puente J, et al. Adherence to the Mediterranean Diet Is Inversely Associated with the Prevalence of Metabolic Syndrome in Older People from the North of Spain. Nutrients.(2022) 14(21), 4536. doi:10.3390/nu14214536

[24] Kim J, Kim M, Shin Y, Cho JH, Lee D, Kim Y. Association between Dietary Diversity Score and Metabolic Syndrome in Korean Adults: A Community-Based Prospective Cohort Study. Nutrients.(2022) 14(24), 5298. doi:10.3390/nu14245298

[25] Szypowska A, Zatońska K, Szuba A, Regulska-Ilow B. Dietary Inflammatory Index (DII)® and Metabolic Syndrome in the Selected Population of Polish Adults: Results of the PURE Poland Sub-Study. Int J Environ Res Public Health.(2023) 20(2), 1056. doi:10.3390/ijerph20021056

[26] Konikowska K, Bombała W, Szuba A, Różańska D, Regulska-Ilow B. A High-Quality Diet, as Measured by the DASH Score, Is Associated with a Lower Risk of Metabolic Syndrome and Visceral Obesity. Biomedicines.(2023) 11(2), 317. doi:10.3390/biomedicines11020317

[27] Shahdadian F, Saneei P, Lotfi K, Feizi A, Askari G, Safavi SM. Association of plant-based diets with adropin, atherogenic index of plasma, and metabolic syndrome and its components: A cross-sectional study on adults. Front Nutr.(2023) 10, 1077709. doi:10.3389/fnut.2023.1077709

[28] Vajdi M, Karimi A, Tousi AZ, Hosseini B, Nikniaz Z, Farhangi MA. Association between plant-based diets and metabolic syndrome in obese adults from Iran: a cross-sectional study. BMC Endocr Disord.(2023) 23(1), 109. doi:10.1186/s12902-023-01358-7

[29] Ghalandari H, Askarpour M, Nouri M, Safarpour AR, Fattahi MR, Akbarzadeh M. Quality of diet and odds of metabolic syndrome in Iranian adults: Baseline results from the PERSIAN Kavar cohort study (PKCS). Nutr Metab Cardiovasc Dis.(2023) 33(9), 1760-1767. doi:10.1016/j.numecd.2023.04.017

[30] Wei L, Fan J, Dong R, Zhang M, Jiang Y, Zhao Q, et al. The Effect of Dietary Pattern on Metabolic Syndrome in a Suburban Population in Shanghai, China. Nutrients.(2023) 15(9), 2185. doi:10.3390/nu15092185

[31] Lanuza F, Meroño T, Zamora-Ros R, Bondonno NP, Rostgaard-Hansen AL, Sánchez-Pla A, et al. Plasma metabolomic profiles of plant-based dietary indices reveal potential pathways for metabolic syndrome associations. Atherosclerosis.(2023) 382, 117285. doi:10.1016/j.atherosclerosis.2023.117285

[32] Muñoz-Cabrejas A, Laclaustra M, Guallar-Castillón P, Casasnovas JA, Marco-Benedí V, Calvo-Galiano N, et al. Low-Quality Carbohydrate Intake Is Associated With a Higher Prevalence of Metabolic Syndrome: The AWHS Study. J Clin Endocrinol Metab.(2024) 109(9), e1768-e1775. doi:10.1210/clinem/dgad706

[33] Farhadnejad H, Mokhtari E, Teymoori F, Jahromi MK, Saber N, Ahmadirad H, et al. Macronutrients quality indices and risk of metabolic syndrome and its components in Iranian adults. BMC Cardiovasc Disord.(2024) 24(1), 126. doi:10.1186/s12872-024-03779-1

[34] Wiese ML, Frost F, Bahls M, von Rheinbaben S, Rühlemann M, Bang C, et al. Dietary Diversity, Rather Than Quality, Parallels a Reduction in Metabolic Syndrome and a Favorable Gut Microbiome: The Dietary Diversity Score. J Am Nutr Assoc.(2025) 44(3), 256-266. doi:10.1080/27697061.2024.2423775

[35] Kang Q, Mei X, Guo C, Si Y, Wang N. Association between Mediterranean diet and metabolic syndrome: analysis of NHANES 2007-2020. Int J Food Sci Nutr.(2025) 76(2), 209-222. doi:10.1080/09637486.2025.2450452

[36] Carvalho CA, Silva AAM, Assunção MCF, Fonseca PCA, Barbieri MA, Bettiol H, et al. The dietary inflammatory index and insulin resistance or metabolic syndrome in young adults. Nutrition.(2019) 58, 187-193. doi:10.1016/j.nut.2018.07.014

[37] Zawdie B, Tesfaye T, Moges SB, Tesfaye Y, Kebede A, Tadesse M, et al. Metabolic syndrome distributions in dietary diversity score groups and its associated factors among adults in the urban community of Jimma, Southwest Ethiopia: a community based cross- sectional study. BMC Endocr Disord.(2022) 22(1), 328. doi:10.1186/s12902-022-01238-6

**Data unavailable (n=19)**

[1] Silva KF, Prata A, Cunha DF. Frequency of metabolic syndrome and the food intake patterns in adults living in a rural area of Brazil. Rev Soc Bras Med Trop.(2011) 44(4), 425-429. doi:10.1590/s0037-86822011000400005

[2] Babio N, Bulló M, Basora J, Martínez-González MA, Fernández-Ballart J, Márquez-Sandoval F, et al. Adherence to the Mediterranean diet and risk of metabolic syndrome and its components. Nutr Metab Cardiovasc Dis.(2009) 19(8), 563-570. doi:10.1016/j.numecd.2008.10.007

[3] Rumawas ME, Meigs JB, Dwyer JT, McKeown NM, Jacques PF. Mediterranean-style dietary pattern, reduced risk of metabolic syndrome traits, and incidence in the Framingham Offspring Cohort. Am J Clin Nutr.(2009) 90(6), 1608-1614. doi:10.3945/ajcn.2009.27908

[4] Paletas K, Athanasiadou E, Sarigianni M, Paschos P, Kalogirou A, Hassapidou M, et al. The protective role of the Mediterranean diet on the prevalence of metabolic syndrome in a population of Greek obese subjects. J Am Coll Nutr.(2010) 29(1), 41-45. doi:10.1080/07315724.2010.10719815

[5] Akbaraly TN, Singh-Manoux A, Tabak AG, Jokela M, Virtanen M, Ferrie JE, et al. Overall diet history and reversibility of the metabolic syndrome over 5 years: the Whitehall II prospective cohort study. Diabetes Care.(2010) 33(11), 2339-2341. doi:10.2337/dc09-2200

[6] Kim MH, Bae YJ. Postmenopausal vegetarians' low serum ferritin level may reduce the risk for metabolic syndrome. Biol Trace Elem Res.(2012) 149(1), 34-41. doi:10.1007/s12011-012-9405-x

[7] Viscogliosi G, Cipriani E, Liguori ML, Marigliano B, Saliola M, Ettorre E, et al. Mediterranean dietary pattern adherence: associations with prediabetes, metabolic syndrome, and related microinflammation. Metab Syndr Relat Disord.(2013) 11(3), 210-216. doi:10.1089/met.2012.0168

[8] Pimenta AM, Toledo E, Rodriguez-Diez MC, Gea A, Lopez-Iracheta R, Shivappa N, et al. Dietary indexes, food patterns and incidence of metabolic syndrome in a Mediterranean cohort: The SUN project. Clin Nutr.(2015) 34(3), 508-514. doi:10.1016/j.clnu.2014.06.002

[9] Farhangi MA, Jahangiry L. Dietary diversity score is associated with cardiovascular risk factors and serum adiponectin concentrations in patients with metabolic syndrome. BMC Cardiovasc Disord.(2018) 18(1), 68. doi:10.1186/s12872-018-0807-3

[10] Fonseca FAH, Izar MCO. Prevalence of Metabolic Syndrome and Framingham Risk Score in Vegetarian and Omnivorous Apparently Healthy Men. Arq Bras Cardiol.(2018) 110(5), 438-439. doi:10.5935/abc.20180083

[11] Khodarahmi M, Asghari-Jafarabadi M, Abbasalizad Farhangi M. A structural equation modeling approach for the association of a healthy eating index with metabolic syndrome and cardio-metabolic risk factors among obese individuals. PLoS One.(2019) 14(7), e0219193. doi:10.1371/journal.pone.0219193

[12] Pandya R, Abdelaal R, Chen JW, Masood S, Talib Z, Atamna H, et al. Retrospective assessment of metabolic syndrome components in early adult life on vegetarian dietary status. Front Public Health.(2022) 10, 945805. doi:10.3389/fpubh.2022.945805

[13] Kaur H, Aeri BT. Metabolic syndrome among apparently healthy adults residing in Delhi: Do vegetarian diets have a protective effect?. J Family Med Prim Care.(2024) 13(1), 143-150. doi:10.4103/jfmpc.jfmpc_340_23

[14] Grant A, Dash C, Adams-Campbell LL. The Association between Diet Quality and Metabolic Syndrome among Older African American Women. Nutrients.(2024) 16(17), 3040. doi:10.3390/nu16173040

[15] Echeverría G, McGee EE, Urquiaga I, Jiménez P, D'Acuña S, Villarroel L, et al. Inverse Associations between a Locally Validated Mediterranean Diet Index, Overweight/Obesity, and Metabolic Syndrome in Chilean Adults. Nutrients.(2017) 9(8), 862. doi:10.3390/nu9080862

[16] Santiago-Torres M, Shi Z, Tinker LF, Lampe JW, Allison MA, Barrington W, et al. Diet quality indices and risk of metabolic syndrome among postmenopausal women of Mexican ethnic descent in the Women's Health Initiative Observational Study. Nutr Healthy Aging.(2020) 5(4), 261-272. doi:10.3233/NHA-190076

[17] Hamedi-Shahraki S, Norouzadeh M, Amirkhizi F. Decreased dietary diversity is a predictor of metabolic syndrome among adults a cross-sectional study in iran. Top Clin Nutr.(2021) 36(4), 272-283. doi:10.1097/TIN.0000000000000264

[18] Sbert PR, Fe BR, Fe NR, Bote SA, González A, Manent J. Relationship between physical activity and adherence to the mediterranean diet with metabolic syndrome, hypertriglyceridemic waist phenotype and hypertensive waist. Med Balear.(2022) 37(6), 33-38. doi:10.3306/AJHS.2022.37.06.33

[19] Jover AM, González A, Gil PT, Villalonga J, Lliteras PM, Manent J. Variables influencing the appearance of metabolic syndrome with three different definitions in 418.343 spanish workers br. Med Balear.(2023) 38(4), 129. doi:10.3306/AJHS.2023.38.04.129

**Meeting summary(n=15)**

[1] Martínez-González MA, Tortosa A, Basterra-Gortari FJ, Bes-Rastrollo M. Mediterranean diet inversely associated with the incidence of metabolic syndrome: the sun prospective cohort response to giugliano, ceriello, and esposito. Diabetes Care.(2008) 31(5), E37. doi:10.2337/dc08-0104

[2] Rizzo NS, Sabate J, Jaceldo K, Fraser G. Vegetarian dietary patterns are associated with a lower risk of metabolic syndrome. The adventist health study 2. Am J Epidemiol.(2010) 171, S71. doi:10.1093/aje/kwq151

[3] Lassale C, Kesse-Guyot E, Fezeu L, Hercberg S, Ahluwalia N, Lairon D. Adherence to mediterranean diet reduces the risk of metabolic syndrome: a prospective study. Ann Nutr Metab.(2011) 58, 57. doi:10.1159/000334393

[4] Ahluwalia N, Lassale C, Hercberg S, Fezeu L, Lairon D, Kesse-Guyot E. Adherence to mediterranean diet assessed using various a priori scores is associated with reduced 6-y risk of metabolic syndrome (mets) in french adults. FASEB J.(2012) 26.

[5] Rizzo N, Sabate J. Dietary patterns and their association with the metabolic syndrome and its component risk factors. Ann Nutr Metab.(2013) 63, 1349. doi:10.1159/000354245

[6] Pérez-Durillo FT, Ramírez M, Villarejo AB, Segarra AB, Prieto I. Anthropometric and nutritional differences between metabolic syndrome patients and a control group. Acta Physiol (Oxf).(2014) 212, 49. doi:10.1111/apha.12388

[7] Khan S, Jackson R. Diet quality and acculturation are associated with metabolic syndrome in females for south asian americans. FASEB J.(2015) 29(1).

[8] Kim HS, Rotundo L, Deutsch J, Kothari N, Ahlawat S, Demyen M, et al. Obesity and cardiovascular disease risk in vegetarians in the united states: a population-based study. Am J Gastroenterol.(2016) 111, S433. doi:10.1038/ajg.2016.360

[9] Echeverría G, Mcgee E, Urquiaga I, Villarroel L, Leighton F, Rigotti A. Adherence to mediterranean diet intake is inversely associated with overweight/ obesity and metabolic syndrome in chilean adult population. Ann Nutr Metab.(2017) 71, 720. doi:10.1159/000480486

[10] Lavigne-Robichaud M, Moubarac JC, Lantagne-Lopez S, Johnson-Down L, Batal M, Laouan Sidi EA, et al. Diet quality indices in relation to metabolic syndrome in an indigenous cree (eeyouch) population in northern québec, canada. Ann Nutr Metab.(2017) 71, 662. doi:10.1159/000480486

[11] Kim H, Rotundo L, Song D, Demyen M, Ahlawat S. The prevalence and characteristics of vegetarian in the united states: a population-based study. Gastroenterology.(2017) 152, S1016.

[12] Yoon Y, Oh S. The association between healthy eating index and metabolic syndrome in korean adults; Analysis of the korea national health and nutrition examination survey (knhanes) 2013-2015. Obes Rev.(2020) 21. doi:10.1111/obr.13118

[13] Cofre SE, Huidobro LA, Ferreccio CR. Inverse association between an index of mediterranean diet and the prevalence of metabolic syndrome in a population of adults aged 38 to 49 years: baseline profile mauco cohort, chile. Clin Nutr ESPEN.(2021) 46, S620. doi:10.1016/j.clnesp.2021.09.222

[14] Abdallah H, Khalil M, Khalil N, Portincasa P, Bonfrate L, Di Ciaula A. Mediterranean diet adherence and metabolic syndrome in lebanese population: a real-time survey study. Eur J Clin Invest.(2022) 52, 28. doi:10.1111/eci.13796

[15] Liu S, Chen B, Jia Y. The association between metabolic syndrome and chinese healthy eating index in a community population of shanghai. Ann Nutr Metab.(2023) 79, 749. doi:10.1159/000530786

**Irrelevant exposure (n=3)**

[1] Motazedian N, Zibaeenezhad MJ, Sayadi M, Khademian F, Hasanzadeh M, Ghorbanpour A, et al. Association of Dietary Patterns with Metabolic Syndrome among Middle-Aged Adults in Shiraz, Iran: Shiraz Heart Study (SHS). J Nutr Metab.(2024) 2024, 1382031. doi:10.1155/2024/1382031

[2] Shakeri Z, Mirmiran P, Khalili-Moghadam S, Hosseini-Esfahani F, Ataie-Jafari A, Azizi F. Empirical dietary inflammatory pattern and risk of metabolic syndrome and its components: Tehran Lipid and Glucose Study. Diabetol Metab Syndr.(2019) 11, 16. doi:10.1186/s13098-019-0411-4

[3] Majdi M, Imani H, Bazshahi E, Hosseini F, Djafarian K, Lesani A, et al. Habitual- and Meal-Specific Carbohydrate Quality Index and Their Relation to Metabolic Syndrome in a Sample of Iranian Adults. Front Nutr.(2022) 9, 763345. doi:10.3389/fnut.2022.763345

**Non-English language (n=2)**

[1] Cornelli U, Belcaro G, Cesarone MR, Di Renz A, Cornelli M, Bamonti F, et al. Monitoring metabolic syndrome in italy: a pilot study. Prog Nutr.(2007) 9(2), 75-87.

[2] Closs VE, Feoli AMP, Schwanke CHA. Metabolic syndrome in elderly from tertiary health care in porto alegre, rio grande do sul, brazil: association with the healthy eating index. Sci Med (Porto Alegre).(2016) 26(3). doi:10.15448/1980-6108.2016.3.23422

**Supplementary Table 3** Characteristics of dietary patterns included in the study.

| **Exposure name** | **Definition/concept** | **Main components** | **No. of studies** |
| --- | --- | --- | --- |
| Mediterranean diet (MD)^[1]^ | The MD refers to the dietary pattern, culture and culinary techniques adhering to countries and populations living in the Mediterranean Sea basin. | A dietary pattern that includes vegetables, fruits, nuts, legumes, whole grains, and olive oil, with moderate amounts of fish, dairy products, and red wine, and limited red meat and processed foods. Fat accounts for 35–45%, mainly from monounsaturated fat, carbohydrate 40–45%, and protein 15–18%. | 9 |
| Dietary Inflammatory Index (DII)^[2]^ | The DII is a literature-derived index designed to quantify the overall pro- and anti-inflammatory potential of an individual’s habitual diet. | The original DII comprises 45 food parameters, including energy; macronutrients; different types of fatty acids; vitamins and minerals; fiber; cholesterol; flavonoids; and selected foods, beverages, herbs, and spices, such as garlic, ginger, onion, pepper, tea, turmeric, and alcohol. | 9 |
| Dietary Approaches to Stop Hypertension (DASH)^[1]^ | The DASH is an evidence-based dietary strategy originally designed to prevent and treat hypertension, which is now extensively applied to assess various cardiometabolic health outcomes. | A dietary pattern with high intake of fruits, vegetables, low-fat dairy products, and whole grains and limited intake of red meat and sugar. Fat accounts for 27% (saturated fat 6%), carbohydrate 55%, and protein 18%. | 6 |
| Oxidative Balance Score (OBS)^[3]^ | The OBS is a composite index designed to assess the overall balance between antioxidant and pro-oxidant exposures from dietary. A higher OBS generally indicates a predominance of antioxidant over pro-oxidant exposures. | Common antioxidant components include dietary fiber, carotenoids, vitamins C and E, folate, selenium, zinc, magnesium, calcium, and other antioxidant nutrients. Common pro-oxidant components include total or saturated fat and iron. Some versions additionally include lifestyle factors such as smoking, alcohol consumption, body mass index, and physical activity. | 6 |
| Healthy Eating Index-related diet quality scores (HEI-related indices)^[4,5]^ | The HEI is an a priori diet quality index developed to assess how closely an individual’s or population’s dietary intake conforms to the Dietary Guidelines for Americans. The Alternative Healthy Eating Index (AHEI) was developed as a modified alternative to the original HEI, with greater emphasis on foods and nutrients that are associated with the risk of chronic diseases. | The HEI rewards sufficient intake of sufficient fruits, vegetables, whole grains, dairy, protein foods, seafood, plant proteins and balanced fatty acids; limited refined grains, sodium, added sugars and saturated fat also gain higher points. The AHEI prioritizes foods and nutrients linked to chronic disease risk. More points are awarded for vegetables, fruits, whole grains, nuts, legumes, long-chain omega-3 and polyunsaturated fats, while less sugar drinks, fruit juice, red/processed meat, trans fat and sodium lead to higher scores. Alcohol is scored separately by fixed intake standards. | 6 |
| Dietary Diversity Score (DDS)^[6]^ | The DDS is an indicator of dietary variety that reflects the number or range of different food groups consumed over a specified reference period and is commonly used as a proxy for overall dietary quality and nutrient adequacy. | The classic DDS is based on five major food groups: grains, vegetables, fruits, dairy products, and meat or other protein-rich foods. Each major food group may be further divided into subgroups according to the scoring method used. Higher scores indicate greater dietary diversity. | 4 |

[1] Castro-Barquero S, Ruiz-León AM, Sierra-Pérez M, Estruch R, Casas R. Dietary Strategies for Metabolic Syndrome: A Comprehensive Review. Nutrients.(2020) 12(10), 2983. doi:10.3390/nu12102983

[2] Vahid F, Goodarzi R, Shivappa N, Hébert JR, Fazeli Moghadam E. Dietary Inflammatory Index (DII®): A significant association between coronary heart disease and DII® in Armenian adults. Eur J Prev Cardiol.(2020) 27(19), 2235-2237. doi:10.1177/2047487319880030

[3] Hernández-Ruiz Á, García-Villanova B, Guerra-Hernández E, Amiano P, Ruiz-Canela M, Molina-Montes E. A Review of A Priori Defined Oxidative Balance Scores Relative to Their Components and Impact on Health Outcomes. Nutrients.(2019) 11(4), 774. doi:10.3390/nu11040774

[4] Krebs-Smith SM, Pannucci TE, Subar AF, Kirkpatrick SI, Lerman JL, Tooze JA, et al. Update of the Healthy Eating Index: HEI-2015. J Acad Nutr Diet.(2018) 118(9), 1591-1602. doi:10.1016/j.jand.2018.05.021

[5] Chiuve SE, Fung TT, Rimm EB, Hu FB, McCullough ML, Wang M, et al. Alternative dietary indices both strongly predict risk of chronic disease. J Nutr.(2012) 142(6), 1009-1018. doi:10.3945/jn.111.157222

[6] Htay ZW, Michihata N, Nakamura Y, Hippo Y, Otonari J, Ikezaki H, et al. Dietary diversity and the risk of metabolic syndrome in a Japanese population: an analysis of baseline cross-sectional data from the J-MICC study. Eur J Clin Nutr.(2025) 79(11), 1106-1113. doi:10.1038/s41430-025-01654-6

**Supplementary Table 4** Quality assessment results of JBI scale in cross-sectional studies.

| **Author, year** | **Were the criteria for inclusion in the sample clearly defined?** | **Were the study subjects and the setting described in detail?** | **Was the exposure measured in a valid and reliable way?** | **Were objective, standard criteria used for measurement of the condition?** | **Were confounding factors identified?** | **Were strategies to deal with confounding factors stated?** | **Were the outcomes measured in a valid and reliable way?** | **Was appropriate statistical analysis used?** |
| --- | --- | --- | --- | --- | --- | --- | --- | --- |
| Panagiotakos et al., 2004 | Yes | Yes | Yes | Yes | Yes | Yes | Yes | Yes |
| Azadbakht et al., 2005 | Yes | Yes | Yes | Yes | Yes | Yes | Yes | Yes |
| A´lvarez Leo´n et al., 2006 | Unclear | Yes | Yes | Yes | Yes | Yes | Yes | Yes |
| Tzima et al., 2009 | Yes | Yes | Yes | Yes | Yes | Yes | Yes | Yes |
| Gouveri et al., 2011 | Unclear | Yes | Yes | Yes | Yes | Yes | Yes | Yes |
| Huang et al., 2011 | Yes | Yes | Yes | Yes | Yes | Yes | Yes | Yes |
| Rizzo et al., 2011 | NO | Yes | Yes | Yes | Yes | Yes | Yes | Yes |
| Saraf-Bank et al., 2017 | Yes | Yes | Yes | Yes | Yes | Yes | Yes | Yes |
| Gholizadeh et al., 2018 | Yes | Yes | Yes | Yes | Yes | Yes | Yes | Yes |
| Kang et al., 2018 | Yes | Yes | Yes | Yes | Yes | Yes | Yes | Yes |
| Kim et al., 2018 | Yes | Yes | Yes | Yes | Yes | Yes | Yes | Yes |
| Nikniaz et al., 2018 | Yes | Yes | Yes | Yes | Yes | Yes | Yes | Yes |
| Ghorabi et al., 2019 | Yes | Yes | Yes | Yes | Yes | Yes | Yes | Yes |
| Phillips et al., 2019 | Yes | Yes | Yes | Yes | Yes | Yes | Yes | Yes |
| Abdollahzad et al., 2020 | Yes | Yes | Yes | Yes | Yes | Yes | Yes | Yes |
| Ariya et al., 2020 | Yes | Yes | Yes | Yes | Yes | Yes | Yes | Yes |
| Ghorabi et al., 2020 | Yes | Yes | Yes | Yes | Yes | Yes | Yes | Yes |
| Amini et al., 2021 | Unclear | Yes | Yes | Yes | Yes | Yes | Yes | Yes |
| Noruzi et al., 2021 | Yes | Yes | Yes | Yes | Yes | Yes | Yes | Yes |
| Zadeh et al., 2021 | Yes | Yes | Yes | Yes | Yes | Yes | Yes | Yes |
| Zadeh et al., 2021 | Yes | Yes | Yes | Yes | Yes | Yes | Yes | Yes |
| Kudsee et al., 2022 | Yes | Yes | Yes | Yes | Yes | Yes | Yes | Yes |
| Li et al., 2022 | Yes | Yes | Yes | Yes | Yes | Yes | Yes | Yes |
| Jovanovic et al., 2023 | Yes | Yes | Yes | Yes | Yes | Yes | Yes | Yes |
| Kahrizsangi et al., 2023 | Yes | Yes | Yes | Yes | Yes | Yes | Yes | Yes |
| Park et al., 2023 | Yes | Yes | Yes | Yes | Yes | Yes | Yes | Yes |
| Xu et al., 2023 | Yes | Yes | Yes | Yes | Yes | Yes | Yes | Yes |
| Gómez-Sánchez et al., 2024 | Yes | Yes | Yes | Yes | Yes | Yes | Yes | Yes |
| Jowshan et al., 2024 | Yes | Yes | Yes | Yes | Yes | Yes | Yes | Yes |
| Li et al., 2024 | Yes | Yes | Yes | Yes | Yes | Yes | Yes | Yes |
| Lu et al., 2024 | Yes | Yes | Yes | Yes | Yes | Yes | Yes | Yes |
| Pashaei et al., 2024 | Yes | Yes | Yes | Yes | Yes | Yes | Yes | Yes |
| Htay et al., 2025 | No | Yes | Yes | Yes | Yes | Yes | Yes | Yes |
| Zhang et al., 2025 | Yes | Yes | Yes | Yes | Yes | Yes | Yes | Yes |

**Supplementary Table 5** GRADE classification of quality of evidence.

| **No.** | **Exposure** | **Outcome** | **Variable type** | **Downgrading for risk of bias** | **Downgrading for Indirecttness** | **Downgrading for inconsistency** | **Downgrading for imprecision** | **Downgrading for publications bias** | **Upgrading for Large effect** | **Upgrading for Dose-response** | **Upgrading for Plausible confounding** | **Quality for GRADE** |
| --- | --- | --- | --- | --- | --- | --- | --- | --- | --- | --- | --- | --- |
| 1 | OBS | Metabolic syndrome | Categorical | Not serious | Not serious | Serious limitation^a^ | Not serious | Not serious | No | No | No | very low |
| 2 | OBS | Metabolic syndrome | Continuous | Not serious | Not serious | Serious limitation^b^ | Not serious | Not serious | No | No | No | very low |
| 3 | MD | Metabolic syndrome | Categorical | Not serious | Not serious | Serious limitation^b^ | Not serious | Not serious | No | No | No | very low |
| 4 | MD | Metabolic syndrome | Continuous | Not serious | Not serious | Serious limitation^a^ | Not serious | Not serious | No | No | No | very low |
| 5 | DII | Metabolic syndrome | Categorical | Not serious | Not serious | Not serious | Not serious | Not serious | No | No | No | low |
| 6 | DASH | Metabolic syndrome | Categorical | Not serious | Not serious | Serious limitation^b^ | Not serious | Not serious | No | No | No | very low |
| 7 | DDS | Metabolic syndrome | Categorical | Not serious | Not serious | Serious limitation^a^ | Not serious | Not serious | No | No | No | very low |
| 8 | HEI | Metabolic syndrome | Categorical | Not serious | Not serious | Serious limitation^a^ | Not serious | Not serious | No | No | No | very low |
| 9 | DII | Abdominal adiposity | Categorical | Not serious | Not serious | Serious limitation^b^ | Not serious | Not serious | No | No | No | very low |
| 10 | OBS | Abdominal adiposity | Categorical | Not serious | Not serious | Serious limitation^b^ | Not serious | Not serious | No | No | No | very low |
| 11 | OBS | Abdominal adiposity | Continuous | Not serious | Not serious | Serious limitation^b^ | Not serious | Not serious | No | No | No | very low |
| 12 | HEI | Abdominal adiposity | Categorical | Not serious | Not serious | Not serious | Not serious | Not serious | No | No | No | low |
| 13 | DII | Low HDL-cholesterol | Categorical | Not serious | Not serious | Serious limitation^b^ | Not serious | Not serious | No | No | No | very low |
| 14 | OBS | Low HDL-cholesterol | Categorical | Not serious | Not serious | Serious limitation^a^ | Not serious | Not serious | No | No | No | very low |
| 15 | OBS | Low HDL-cholesterol | Continuous | Not serious | Not serious | Not serious | Not serious | Not serious | No | No | No | low |
| 16 | HEI | Low HDL-cholesterol | Categorical | Not serious | Not serious | Serious limitation^a^ | Not serious | Not serious | No | No | No | very low |
| 17 | DII | High serum triglyceride | Categorical | Not serious | Not serious | Serious limitation^b^ | Not serious | Not serious | No | No | No | very low |
| 18 | OBS | High serum triglyceride | Categorical | Not serious | Not serious | Not serious | Not serious | Not serious | No | No | No | low |
| 19 | OBS | High serum triglyceride | Continuous | Not serious | Not serious | Serious limitation^b^ | Not serious | Not serious | No | No | No | very low |
| 20 | HEI | High serum triglyceride | Categorical | Not serious | Not serious | Not serious | Not serious | Not serious | No | No | No | low |
| 21 | DII | Abnormal glucose homeostasis | Categorical | Not serious | Not serious | Not serious | Not serious | Not serious | No | No | No | low |
| 22 | OBS | Abnormal glucose homeostasis | Categorical | Not serious | Not serious | Not serious | Not serious | Not serious | No | No | No | low |
| 23 | OBS | Abnormal glucose homeostasis | Categorical | Not serious | Not serious | Not serious | Not serious | Not serious | No | No | No | low |
| 24 | HEI | Abnormal glucose homeostasis | Categorical | Not serious | Not serious | Not serious | Not serious | Not serious | No | No | No | low |
| 25 | DII | Elevated blood pressure | Categorical | Not serious | Not serious | Not serious | Not serious | Not serious | No | No | No | low |
| 26 | OBS | Elevated blood pressure | Categorical | Not serious | Not serious | Not serious | Not serious | Not serious | No | No | No | low |
| 27 | OBS | Elevated blood pressure | Continuous | Not serious | Not serious | Serious limitation^a^ | Not serious | Not serious | No | No | No | very low |
| 28 | HEI | Elevated blood pressure | Categorical | Not serious | Not serious | Serious limitation^a^ | Not serious | Not serious | No | No | No | very low |

Abbreviations: OBS, Oxidative Balance Score; MD, Mediterranean Diet; DII, Dietary Inflammatory Index; DASH, Dietary Approaches to Stop Hypertension; DDS, Dietary Diversity Score; HEI, Healthy Eating Index.

Notes: Blue text indicates downgrade items; red text indicates upgrade items.

^a^ Downgraded by one level for inconsistency: substantial heterogeneity is seen between studies (I2 > 50%).

^b^ Downgraded by two levels for inconsistency: substantial heterogeneity is seen between studies (I2 > 75%), whereas heterogeneity was unexplained.

**Supplementary Table 6** Pooled associations between dietary patterns and five components of metabolic syndrome.

| **Number** | **Exposure** | **Outcome** | **Variable type** | **Number of studies** | **OR (95%CI)** | **P** | **I^2^** |
| --- | --- | --- | --- | --- | --- | --- | --- |
| 1 | DII | abdominal adiposity | Categorical | 8 | 1.49 (1.12, 2.00) | 0.007 | 89.4% |
| 2 | HEI | abdominal adiposity | Categorical | 3 | 0.85 (0.70, 1.03) | 0.093 | 0.0% |
| 3 | OBS | abdominal adiposity | Categorical | 5 | 0.40 (0.27, 0.61) | < 0.001 | 94.7% |
| 4 | OBS | abdominal adiposity | Continuous | 3 | 0.94 (0.92, 0.95) | < 0.001 | 84.6% |
| 5 | DII | low HDL-cholesterol | Categorical | 8 | 1.24 (1.00, 1.52) | 0.047 | 84.6% |
| 6 | HEI | low HDL-cholesterol | Categorical | 3 | 0.91 (0.70, 1.17) | 0.439 | 71.6% |
| 7 | OBS | low HDL-cholesterol | Categorical | 5 | 0.62 (0.52, 0.74) | < 0.001 | 61.2% |
| 8 | OBS | low HDL-cholesterol | Continuous | 3 | 0.96 (0.96, 0.97) | < 0.001 | 22.2% |
| 9 | DII | high serum triglyceride | Categorical | 8 | 1.16 (0.95, 1.42) | 0.154 | 86.1% |
| 10 | HEI | high serum triglyceride | Categorical | 3 | 0.74 (0.61, 0.88) | 0.001 | 0.0% |
| 11 | OBS | high serum triglyceride | Categorical | 5 | 0.63 (0.55, 0.72) | < 0.001 | 41.5% |
| 12 | OBS | high serum triglyceride | Continuous | 3 | 0.97 (0.95, 0.98) | < 0.001 | 80.6% |
| 13 | DII | abnormal glucose homeostasis | Categorical | 8 | 1.19 (1.12, 1.26) | < 0.001 | 0.0% |
| 14 | HEI | abnormal glucose homeostasis | Categorical | 3 | 0.82 (0.68, 0.99) | 0.041 | 0.0% |
| 15 | OBS | abnormal glucose homeostasis | Categorical | 5 | 0.70 (0.63, 0.78) | < 0.001 | 0.0% |
| 16 | OBS | abnormal glucose homeostasis | Continuous | 3 | 0.98 (0.97, 0.98) | < 0.001 | 11.6% |
| 17 | DII | elevated blood pressure | Categorical | 7 | 1.25 (1.18, 1.32) | < 0.001 | 0.0% |
| 18 | HEI | elevated blood pressure | Categorical | 3 | 0.79 (0.52, 1.19) | 0.260 | 71.7% |
| 19 | OBS | elevated blood pressure | Categorical | 4 | 0.61 (0.54, 0.70) | < 0.001 | 12.4% |
| 20 | OBS | elevated blood pressure | Continuous | 3 | 0.97 (0.96, 0.98) | < 0.001 | 74.0% |

Abbreviations: OBS, Oxidative Balance Score; MD, Mediterranean Diet; DII, Dietary Inflammatory Index; DASH, Dietary Approaches to Stop Hypertension; DDS, Dietary Diversity Score; HEI, Healthy Eating Index.

**Supplementary Table 7** Subgroup analyses stratified by sex for associations between dietary patterns and metabolic syndrome and its components.

| **Number** | **Exposure** | **Outcome** | **Variable type** | **Subgroup** | **Number of studies** | **OR (95%CI)** | **P** | I**^2^** |
| --- | --- | --- | --- | --- | --- | --- | --- | --- |
| 1 | DII | Metabolic syndrome | Categorical | female | 3 | 1.37 (1.22, 1.54) | < 0.001 | 0.0% |
| 2 | DII | Metabolic syndrome | Categorical | male | 3 | 1.34 (1.08, 1.66) | 0.007 | 50.1% |
| 3 | OBS | Metabolic syndrome | Categorical | female | 4 | 0.40 (0.26, 0.61) | < 0.001 | 88.3% |
| 4 | OBS | Metabolic syndrome | Categorical | male | 4 | 0.53 (0.47, 0.60) | < 0.001 | 0.0% |
| 5 | OBS | Metabolic syndrome | Continuous | female | 4 | 0.91 (0.88, 0.95) | < 0.001 | 93.1% |
| 6 | OBS | Metabolic syndrome | Continuous | male | 4 | 0.93 (0.90, 0.96) | < 0.001 | 91.8% |
| 7 | MD | Metabolic syndrome | Categorical | female | 3 | 0.75 (0.41, 1.36) | 0.344 | 89.9% |
| 8 | MD | Metabolic syndrome | Categorical | male | 3 | 0.78 (0.57, 1.07) | 0.120 | 62.9% |
| 9 | HEI | Metabolic syndrome | Categorical | female | 3 | 0.81 (0.66, 1.01) | 0.057 | 0.0% |
| 10 | DII | Abdominal adiposity | Categorical | female | 3 | 1.45 (1.30, 1.61) | < 0.001 | 0.0% |
| 11 | DII | Abdominal adiposity | Categorical | male | 3 | 1.32 (1.13, 1.53) | < 0.001 | 15.1% |
| 12 | DII | Low HDL-cholesterol | Categorical | female | 3 | 1.19 (0.77, 1.84) | 0.444 | 93.0% |
| 13 | DII | Low HDL-cholesterol | Categorical | male | 3 | 1.20 (0.87, 1.66) | 0.267 | 79.4% |
| 14 | DII | High serum triglyceride | Categorical | female | 3 | 1.27 (1.14, 1.42) | < 0.001 | 20.8% |
| 15 | DII | High serum triglyceride | Categorical | male | 3 | 1.19 (1.06, 1.33) | 0.004 | 7.1% |
| 16 | DII | Abnormal glucose homeostasis | Categorical | female | 3 | 1.25 (0.97, 1.63) | 0.091 | 80.5% |
| 17 | DII | Abnormal glucose homeostasis | Categorical | male | 3 | 1.29 (0.94, 1.75) | 0.114 | 83.6% |
| 18 | HEI | Abnormal glucose homeostasis | Categorical | female | 3 | 0.80 (0.62, 1.02) | 0.073 | 0.0% |
| 19 | DII | Elevated blood pressure | Categorical | female | 3 | 1.28 (1.18, 1.38) | < 0.001 | 0.9% |
| 20 | DII | Elevated blood pressure | Categorical | male | 3 | 1.19 (1.08, 1.32) | 0.001 | 0.0% |

Abbreviations: OBS, Oxidative Balance Score; MD, Mediterranean Diet; DII, Dietary Inflammatory Index; DASH, Dietary Approaches to Stop Hypertension; DDS, Dietary Diversity Score; HEI, Healthy Eating Index.

**Supplementary Table 8** Subgroup analyses stratified by study design for associations between dietary patterns and metabolic syndrome and its components.

| **Number** | **Exposure** | **Outcome** | **Variable type** | **Subgroup** | **Number of studies** | **OR (95%CI)** | **P** | **I^2^** |
| --- | --- | --- | --- | --- | --- | --- | --- | --- |
| 1 | DII | Metabolic syndrome | Categorical | Cross sectional | 6 | 1.33 (1.21, 1.46) | < 0.001 | 0.0% |
| 2 | OBS | Metabolic syndrome | Categorical | Cross sectional | 6 | 0.46 (0.38, 0.56) | < 0.001 | 67.1% |
| 3 | OBS | Metabolic syndrome | Continuous | Cross sectional | 4 | 0.95 (0.94, 0.96) | < 0.001 | 73.6% |
| 4 | MD | Metabolic syndrome | Categorical | Cross sectional | 6 | 0.82 (0.66, 1.02) | 0.080 | 79.6% |
| 5 | DASH | Metabolic syndrome | Categorical | Cross sectional | 3 | 0.51 (0.28, 0.93) | 0.028 | 68.70% |
| 6 | DDS | Metabolic syndrome | Categorical | Cross sectional | 3 | 0.74 (0.53, 1.03) | 0.078 | 80.4% |
| 7 | HEI | Metabolic syndrome | Categorical | Cross sectional | 4 | 0.82 (0.64, 1.06) | 0.123 | 66.8% |
| 8 | DII | Abdominal adiposity | Categorical | Cross sectional | 6 | 1.43 (0.96, 2.12) | 0.083 | 90.0% |
| 9 | DII | Low HDL-cholesterol | Categorical | Cross sectional | 6 | 1.15 (0.94, 1.42) | 0.182 | 78.3% |
| 10 | DII | High serum triglyceride | Categorical | Cross sectional | 6 | 1.08 (0.84, 1.40) | 0.554 | 86.0% |
| 11 | DII | Abnormal glucose homeostasis | Categorical | Cross sectional | 6 | 1.21 (1.07, 1.37) | 0.002 | 19.2% |
| 12 | DII | Elevated blood pressure | Categorical | Cross sectional | 5 | 1.24 (1.14, 1.36) | < 0.001 | 0.0% |

Abbreviations: OBS, Oxidative Balance Score; MD, Mediterranean Diet; DII, Dietary Inflammatory Index; DASH, Dietary Approaches to Stop Hypertension; DDS, Dietary Diversity Score; HEI, Healthy Eating Index.

**Supplementary Table 9** Subgroup analyses stratified by continent for associations between dietary patterns and metabolic syndrome and its components.

| **Number** | **Exposure** | **Outcome** | **Variable type** | **Subgroup** | **Number of studies** | **OR (95%CI)** | **P** | **I^2^** |
| --- | --- | --- | --- | --- | --- | --- | --- | --- |
| 1 | DII | Metabolic syndrome | Categorical | Asian | 7 | 1.32 (1.23, 1.43) | < 0.001 | 0.0% |
| 2 | OBS | Metabolic syndrome | Categorical | Asian | 3 | 0.54 (0.39, 0.75) | < 0.001 | 79.9% |
| 3 | OBS | Metabolic syndrome | Categorical | North American | 4 | 0.44 (0.36, 0.55) | < 0.001 | 64.9% |
| 4 | OBS | Metabolic syndrome | Continuous | North American | 3 | 0.95 (0.95, 0.96) | < 0.001 | 22.2% |
| 5 | MD | Metabolic syndrome | Categorical | European | 5 | 0.80 (0.64, 1.01) | 0.059 | 82.7% |
| 6 | DASH | Metabolic syndrome | Categorical | Asian | 3 | 0.65 (0.35, 1.20) | 0.167 | 84.1% |
| 7 | HEI | Metabolic syndrome | Categorical | Asian | 4 | 0.74 (0.60, 0.89) | 0.002 | 15.9% |
| 8 | DII | Abdominal adiposity | Categorical | Asian | 7 | 1.44 (1.06, 1.94) | 0.019 | 90.7% |
| 9 | OBS | Abdominal adiposity | Categorical | North American | 4 | 0.38 (0.24, 0.60) | < 0.001 | 95.9% |
| 10 | DII | Low HDL-cholesterol | Categorical | Asian | 7 | 1.24 (1.00, 1.53) | 0.052 | 86.8% |
| 11 | OBS | Low HDL-cholesterol | Categorical | North American | 4 | 0.58 (0.52, 0.64) | < 0.001 | 0.0% |
| 12 | DII | High serum triglyceride | Categorical | Asian | 7 | 1.11 (0.91, 1.36) | 0.307 | 87.1% |
| 13 | OBS | High serum triglyceride | Categorical | North American | 4 | 0.61 (0.53, 0.70) | < 0.001 | 41.4% |
| 14 | DII | Abnormal glucose homeostasis | Categorical | Asian | 7 | 1.19 (1.12, 1.27) | < 0.001 | 4.7% |
| 15 | OBS | Abnormal glucose homeostasis | Categorical | North American | 4 | 0.70 (0.62, 0.78) | < 0.001 | 0.0% |
| 16 | DII | Elevated blood pressure | Categorical | Asian | 6 | 1.24 (1.17, 1.32) | < 0.001 | 0.0% |

Abbreviations: OBS, Oxidative Balance Score; MD, Mediterranean Diet; DII, Dietary Inflammatory Index; DASH, Dietary Approaches to Stop Hypertension; DDS, Dietary Diversity Score; HEI, Healthy Eating Index.

**Supplementary Figure 1** Leave-one-out sensitivity analysis of DASH, DDS, DII, and HEI with MetS.

Abbreviations: DASH, Dietary Approaches to Stop Hypertension; DDS, Dietary Diversity Score; DII, Dietary Inflammatory Index; HEI, Healthy Eating Index; MetS, Metabolic Syndrome.

**Supplementary Figure 2** Leave-one-out sensitivity analysis of MD and OBS with MetS.

Abbreviations: MD, Mediterranean Diet; OBS, Oxidative Balance Score; MetS, Metabolic Syndrome.

**Supplementary Figure 3** Leave-one-out sensitivity analysis of DII, HEI, and OBS with abdominal adiposity.

Abbreviations: DII, Dietary Inflammatory Index; HEI, Healthy Eating Index; OBS, Oxidative Balance Score.

**Supplementary Figure 4** Leave-one-out sensitivity analysis of DII, HEI, and OBS with low HDL-cholesterol.

Abbreviations: DII, Dietary Inflammatory Index; HEI, Healthy Eating Index; OBS, Oxidative Balance Score.

**Supplementary Figure 5** Leave-one-out sensitivity analysis of DII, HEI, and OBS with high serum triglyceride.

Abbreviations: DII, Dietary Inflammatory Index; HEI, Healthy Eating Index; OBS, Oxidative Balance Score.

**Supplementary Figure 6** Leave-one-out sensitivity analysis of DII, HEI, and OBS with abnormal glucose homeostasis.

Abbreviations: DII, Dietary Inflammatory Index; HEI, Healthy Eating Index; OBS, Oxidative Balance Score.

**Supplementary Figure 7** Leave-one-out sensitivity analysis of DII, HEI, and OBS with elevated blood pressure.

Abbreviations: DII, Dietary Inflammatory Index; HEI, Healthy Eating Index; OBS, Oxidative Balance Score.
